# The oral maternal microbiome plays a role in the development of cleft lip and palate condition in children

**DOI:** 10.7717/peerj.21128

**Published:** 2026-04-27

**Authors:** Suzana Eiko Sato Guima, Bárbara Bischain, Lívia C. Morais Gama, Agatha Cristhina Faria, Talita Lourenço, Daniela Franco Bueno, Debora Heller, Maria Rita Passos-Bueno, João C. Setubal

**Affiliations:** 1 Departamento de Bioquímica, Instituto de Química, Universidade de São Paulo, São Paulo, Brazil; 2 Centro de Estudos do Genoma Humano, Departamento de Genética e Biologia Evolutiva, Instituto de Biociências, Universidade de São Paulo, São Paulo, Brazil; 3 Instituto de Microbiologia Paulo de Góes, Departamento de Microbiologia Médica, Universidade Federal do Rio de Janeiro, Rio de Janeiro, Brazil; 4 Albert Einstein Israeli College of Health Sciences, Albert Einstein Hospital, São Paulo, Brazil; 5 UT Health San Antonio, University of Texas, San Antonio, TX, United States of America; 6 Universidade Cruzeiro do Sul, São Paulo, Brazil

**Keywords:** Microbiome, rRNA 16S unit, Non-syndromic cleft lip or palate, Maternal oral microbiome, Male babies, *Cutibacterium*, *Limosilactobacillus*

## Abstract

Non-syndromic cleft lip or palate (NS-CL/P) is an oral birth defect with complex aetiology. We compared the microbial diversity and composition of the oral microbiome of mothers of babies with NS-CL/P (CLP group) and mothers of babies without NS-CL/P (control group). Oral microbiome composition was determined by sequencing the V3-V4 regions of the 16S rRNA gene. CLP and control groups had overall similar microbial compositions, but significant differences were observed. The most significant microbial genus related to these differences was *Cutibacterium*, which was more abundant in the CLP group. Based on the literature, we hypothesize that a member of the *Cutibacterium* genus present in the oral microbiota may have a role in inflammation processes that could be related to NS-CL/P development. We found additional differences in terms of differential abundance when subsetting the dataset for mothers with a male child; in this case, depletion of *Limosilactobacillus* and an unknown taxon, in the CLP group, was a significant result. We conclude that the maternal oral microbiome likely plays a role in the development of the NS-CL/P condition.

## Introduction

One of the most common craniofacial malformations is the cleft lip with or without cleft palate (CL/P), with an average prevalence of 1:1000 births (Salari et al., 2022). Most cases are classified as non-syndromic CL/P (NS-CL/P), where the oral cleft is the only malformation (Dixon et al., 2011). Ancestry and socio-economic factors significantly contribute to the variability of prevalence estimates across different populations. Families with affected children have to deal with speech therapy, dental treatment, surgeries, post-surgical care, psychosocial intervention, and consequently, financial risk and social burden (Wehby & Cassell, 2010).

CL/P has a complex inheritance model, meaning that the oral defect can be caused by a combination of genetic, environmental, and maternal lifestyle factors (Dixon et al., 2011; Nasreddine, El Hajj & Ghassibe-Sabbagh, 2021). The heritability of CL/P varies from 45 to 85% depending on the population, suggesting the relevance of genetic factors in its aetiology (Brito et al., 2011). A substantial worldwide effort has been made to dissect the genetic architecture of CL/P by analysis of common variants using Genome-Wide Association Studies (GWAS). Most recently, some studies using exome or genome sequencing (whole exome sequencing (WES) or whole genome sequencing (WGS)) showed that rare variants contribute to a proportion of CL/P cases (Dixon et al., 2011; Brito et al., 2015; Nasreddine, El Hajj & Ghassibe-Sabbagh, 2021).

Several environmental factors have been associated with the risk of CL/P, including low socio-economic status (Ferrari-Piloni et al., 2021; Ács et al., 2024), smoking (Vieira et al., 2021; Xu et al., 2021; Yang et al., 2022; Vathulya et al., 2024), nutritional factors (Yoshida et al., 2020), folic acid deficiency (Xu et al., 2021), obesity (Kutbi et al., 2017), maternal infections (Divya, 2017; Ács et al., 2024), and periodontal disease (Bánhidy et al., 2010). Among these, infections and periodontal diseases presented the strongest associations with oral clefts, with reported odds ratios exceeding 4.0. Periodontal disorders, which affect up to 40% or even higher percentages in pregnant women, are associated with maternal oral microbiome dysbiosis, which in turn have been linked to adverse pregnancy outcomes (Ye & Kapila, 2021; Saadaoui, Singh & Al Khodor, 2021). A proposed mechanism underlying this process is the activation of pro-inflammatory responses triggered by systemic dissemination of oral bacteria, as transient bacteremia can be induced by routine tooth brushing (Costalonga & Herzberg, 2014; Saadaoui, Singh & Al Khodor, 2021; Baker et al., 2024). Comparable mechanisms have been implicated in cancer, diabetes, and Alzheimer’s disease (Hajishengallis & Chavakis, 2021). Although the presence of bacteria in the placenta remains controversial (Kennedy et al., 2023), several studies have demonstrated that increased maternal levels of pro-inflammatory molecules can contribute to placenta inflammation (Challier et al., 2008; Couture et al., 2023). Recent experimental findings have strengthened the connection between maternal inflammation and genetic susceptibility to CL/P. In *in vitro* (human induced pluripotent cells-iPSC) and *in vivo* models (*Xenopus*, mouse) deficient in E-cadherin (CDH1), a recognized genetic risk factor for CL/P, exposure to pro-inflammatory molecules induced methylation of the CDH1 promoter, which was associated with the occurrence of oral clefts in mice (Alvizi et al., 2017; Alvizi et al., 2023). Taken together, these observations underscore the multifactorial nature of CL/P, and suggest that maternal oral dysbiosis during pregnancy may represent an important, yet underexplored, environmental risk for CL/P.

Our goal with the present work was to determine whether the oral microbiome of mothers has a role in the development of NS-CL/P in their babies. To test the hypothesis that the oral microbiome does have a role, we compared the oral microbiome of mothers who had babies with NS-CL/P with that from mothers who did not, using 16S rRNA metabarcoding. The data and our analyses suggest that indeed the maternal oral microbiome has a role in NS-CL/P development, although the evidence is subtle and the identification of the microbial species involved requires additional work.

## Methods

*Ethics*. The study was approved by the University of São Paulo, Menino Jesus Hospital (3.045.972), and Albert Einstein Hospital (CAAE: 43543615.8.3004.0071) ethics committees; the ethics committee approval for the Albert Einstein Hospital also covers the Santa Catarina Municipality Hospital. The experimental procedures were conducted according to the Declaration of Helsinki. All participants provided written informed consent before any assessment (Supplementary Information Files).

*Participants*. Mothers of babies with NS-CL/P (*n* = 34) (the *CLP group*) and mothers of babies with normal lip and palate (*n* = 36) (the *control group*) were recruited at Menino Jesus Hospital and at Santa Catarina Municipality Hospital, respectively (both in São Paulo city, Brazil). The healthcare provided in these hospitals is under the Sistema Unificado de Saúde (SUS) program, which is a public health program that serves low and medium-low-income populations. Mothers who had babies with or without NS-CL/P associated with other dysmorphisms or congenital malformations were not included.

*Demographics Assessment.* At the time of sample collection, a questionnaire was completed with data about the health history and habits of the study participants, before and during pregnancy (Supplementary Document S1).

*Oral health examination.* Oral health examination was performed by two calibrated dentists. The Modified Gingival Index (MGI) was applied as a non-invasive visual scale to assess the presence of gingival inflammation. Five categories, using a 0-4 scale, score the marginal and papillary gingival tissue based on color, texture, edema, and spontaneous bleeding (Lobene et al., 1986).

*Sample collection.* Samples were collected over a five-year period (2015–2019) as follows: CLP group: February 2015–June 2018; control group: June 2018–March 2019. In order to ensure comparable socio-economic backgrounds, collections were carried out in public SUS hospitals (see above). Although legally accessible by anyone, SUS hospitals are predominantly used by lower- and middle-income groups. Hospital Menino Jesus, a municipal pediatric reference center for CL/P in São Paulo, concentrates most CL/P cases in the city; however, unexpectedly, the number of non-CL/P newborns of comparable age to the CL/P newborns was insufficient (one case in one year). Therefore, we established a collaboration with Hospital Santa Catarina, a SUS maternity referral hospital, also in São Paulo city, for control sample collection.

Whole saliva samples were collected by research team members *via* passive drooling using the OMNIgene-Discover (OM-501) collection kit. This kit has a saliva collection system that stabilizes microbial DNA by preserving the sample at room temperature for up to one year. After collection, the samples were transported to the Human Genomics and Stem-Cell Study Center at the University of São Paulo for DNA extraction following the manufacturer’s instructions. Saliva samples were stored at −20 °C. DNA was extracted up to two months after sample collection.

*DNA extraction, amplification, and sequencing for microorganisms’ detection.* Microbial DNA was extracted with DNeasy PowerBiofilm Kit (Qiagen, Hilden Germany) and quantified using a NanoDrop spectrophotometer (Thermo Scientific, Waltham, MA, USA). The composition of the oral microbiome was determined by sequencing (Illumina Miseq platform, 300 bp, paired-end) of the V3-V4 hypervariable regions of the 16S rRNA gene (Table S1), and the raw data was deposited in the Sequence Read Archive (PRJNA1066742).

*Metadata analysis.* In order to verify whether there were characteristics between both groups that could be confounding factors, we tested the null hypothesis of equal rank sum between the CLP and control groups for the numeric variables using the Wilcoxon rank sum test. This non-parametric analysis was used based on the assumption of normal distribution, analyzed with the Shapiro–Wilk test (Table S2). The null hypothesis for the Wilcoxon test was rejected if the *p*-value was less than 0.05. The alternative hypothesis is a different rank sum between CLP and control groups.

If a difference in the oral microbiome is found between CLP and control groups in the downstream analysis, there is the possibility of an associated variable influencing this difference. To check whether a categorical variable in this study was associated with NS-CL/P, we tested the null hypothesis of non-association using Pearson’s Chi-squared test. Fisher’s Exact test was used in cases where one cell of the contingency table had a count of equal to or fewer than four. The null hypothesis of non-association was rejected if the *p*-value was less than 0.05. The alternative hypothesis was an association between the categorical variable in analysis and NS-CL/P.

*Sequence data analysis.* We performed the following procedures using plugins from the QIIME2 pipeline v.2023.5 (Bolyen et al., 2019). Raw sequence data had adapters trimmed using cutadapt (Martin, 2011), discarding all untrimmed sequences (“–p-front-f CCTACGGGNGGCWGCAG –p-front-r GACTACHVGGGTATCTAATCC”). DADA2 (Callahan et al., 2016) was used to infer amplicon sequence variants (ASVs), truncating forward reads at 230 bp and reverse reads at 227 bp, in order to remove low-quality regions. The DADA2 pipeline uses an error model to infer and correct sequencing errors, merge read pairs, and remove putative PCR chimeras. We then removed possibly spurious ASVs by filtering out ASVs found in only one sample (71 singletons). As a result, we obtained a table with the counts of ASVs per sample and the representative sequence of each ASV from the output of DADA2.

For the taxonomic classification of ASVs, we mapped ASVs to the Greengenes2 database (v.2024.09) (McDonald et al., 2024) using the “greengenes2 non-v4-16s” method in the “q2-greengenes2” QIIME2 plugin (McDonald, 2023). This generated a new feature table with taxonomic assignments. (The Greengenes2 reference tree and taxonomy are derived from ∼380 global marker genes and full-length 16S rRNA sequences).

To account for variable sequencing depth (Cameron et al., 2021), we performed repeated rarefaction (100 iterations at a depth of 31,000 sequences per sample) using the “q2-repeat-rarefy” plugin (Yao, 2021). This depth was chosen as the read count of the second-smallest sample to maximize data retention. Consequently, one sample (S53) with only 27,243 reads was excluded from all rarefaction-based analyses. The rarefied table was used to calculate alpha diversity metrics (Observed features, Pielou’s Evenness (Pielou, 1966), Shannon (Shannon, 1948), Simpson (Simpson, 1949), and Faith Phylogenetic Diversity (Faith, 1992) and beta diversity distances (Bray-Curtis (Sørensen, 1948), Jaccard (Jaccard, 1908), Unweighted Unifrac (Lozupone & Knight, 2005), and Weighted Unifrac (Lozupone et al., 2007). The phylogenetic diversity metrics utilized the Greengenes2 reference tree. In contrast, Aitchison distance (Aitchison, 1982)—a metric based on centered log-ratio (CLR) transformation recommended for compositional data (Gloor et al., 2017)—was calculated from the unrarefied table, thereby including sample S53.

We compared the alpha diversity between the CLP and control groups using the Kruskal–Wallis rank sum test (Kruskal & Wallis, 1952). Beta diversity was compared using PERMANOVA (Anderson, 2001) (“adonis2” in the R vegan package (Jari Oksanen et al., 2018)) on distance matrices, visualized *via* Principal Coordinate Analysis (PCoA) (Halko et al., 2011) with group ellipses (“ggplot2::stat_ellipse” (Friendly, Monette & Fox, 2013; Fox & Weisberg, 2018)).

Differential abundance analysis was performed using ANCOM-BC *v.* 2.6.0 (Lin & Peddada, 2020) on the non-rarefied (absolute abundance) ASV table at each taxonomic level. The model controlled for the following covariates: child’s sex, maternal urinary infection, gingivitis, caries, gestational hypertension, antibiotic usage, and child age at sampling, with control as the reference group. We used default parameters with a prevalence cutoff (prv_cut) of 0.10 and considered results with a Benjamini–Hochberg adjusted *p*-value < 0.05 as significant.

We predicted metagenomic functions from the ASV table using the standard PICRUSt2 workflow (version 2.6.3, “picrust2_pipeline.py–min_samples 7–stratified–per_sequence_contrib”) (Ye & Doak, 2009; Louca & Doebeli, 2018; Barbera et al., 2019; Douglas et al., 2020) and assessed functional enrichment between groups with ALDEx2 (default parameters, *t*-test, 128 Monte Carlo samples). Predicted MetaCyc pathways (Caspi et al., 2018) were annotated (top and second-level categories) using publicly available mapping files at Jiung-Wen’s GitHub (Chen, 2018).

We repeated all alpha/beta diversity analyses on data stratified by the following covariates: infant sex, maternal gingivitis status, caries status, hypertensive status, antibiotic use, and urinary infection status. Due to low sample sizes, we did not stratify analysis by maternal periodontitis, smoking, or diabetes status. Within each stratum, CLP and control groups were compared using the same statistical methods applied to the full dataset. For strata where a significant difference was observed in at least three alpha or beta diversity metrics, we proceeded with differential abundance analysis using ANCOM-BC, following the same method as for the unstratified data. This criterion was met for the following subsets: mothers of male infants, those without urinary infection, those with gingivitis, non-hypertensive participants, and those without antibiotic use. Finally, we performed functional prediction (PICRUSt2 and ALDEx2) for any stratum where differential abundance analysis identified at least one differentially abundant taxon that was not significant in the full-sample comparison.

## Results

### Participant characteristics and clinical data

The study included 70 mothers: 34 mothers of neonates with NS-CL/P (CLP group) and 36 mothers of unaffected neonates (control group) without a first-degree related CL/P individual. Maternal age was similar between groups (CLP: 26.94 ± 6.81 years; control: 27.75 ± 7.41 years). Descriptive statistics for all sociodemographic, general health, and oral health variables are provided in Table 1.

**Table 1:** Characteristics of study participants.

**Variable**	**CLP**	**Control**	**Total**
n (sample size)	34	36	70
Maternal age in years (mean ± SD)	26.94 ± 6.81	27.75 ± 7.41	27.37 ± 7.09
Median [min, max]	26.5 [16, 46]	26.5 [15, 42]	26.5 [15, 46]
Gingivitis			
No (n, %)	28 (82.4%)	21 (61.8%)	49 (72.1%)
Yes (n, %)	6 (17.6%)	13 (38.2%)	19 (27.9%)
Periodontitis			
No (n, %)	31 (91.2%)	32 (94.1%)	63 (92.6%)
Yes (n, %)	3 (8.8%)	2 (5.9%)	5 (7.4%)
Caries			
No (n, %)	22 (64.7%)	27 (81.8%)	49 (73.1%)
Yes (n, %)	12 (35.3%)	6 (18.2%)	18 (26.9%)
Smoking during gestation			
No (n, %)	28 (82.4%)	36 (100.0%)	64 (91.4%)
Yes (n, %)	6 (17.6%)	0 (0.0%)	6 (8.6%)
Gestational hypertension			
No (n, %)	29 (85.3%)	25 (69.4%)	54 (77.1%)
Yes (n, %)	5 (14.7%)	11 (30.6%)	16 (22.9%)
Gestational diabetes			
No (n, %)	31 (91.2%)	35 (97.2%)	66 (94.3%)
Yes (n, %)	3 (8.8%)	1 (2.8%)	4 (5.7%)
Urinary infection			
No (n, %)	19 (57.6%)	18 (51.4%)	37 (54.4%)
Yes (n, %)	14 (42.4%)	17 (48.6%)	31 (45.6%)
Antibiotic usage			
No (n, %)	16 (47.1%)	25 (69.4%)	41 (58.6%)
Yes (n, %)	18 (52.9%)	11 (30.6%)	29 (41.4%)
**Children’s characteristics**			
Sex			
F (n, %)	7 (23.3%)	15 (44.1%)	22 (33.8%)
M (n, %)	23 (76.7%)	20 (57.1%)	43 (66.2%)
Weeks of gestation (mean ± SD)	37.87 ± 3.12	38.90 ± 1.92	38.38 ± 2.63
Median [min, max]	39 [24, 41]	40 [32, 41]	39 [24, 41]
Days postpartum and child’s age during maternal sampling in days (mean ± SD)	22.91 ± 20.31	2.43 ± 5.31	12.21 ± 17.73
Median [min, max]	14.5 [1, 81]	1 [0, 30]	2 [0, 81]

**Notes.**

CLP Cleft lip and palate group n number of samples SD standard deviation min minimum value max maximum value % relative abundance

The CLP and control groups were generally similar in terms of maternal characteristics. No significant differences were found in maternal age or weeks of gestation (*p*-value > 0.05). Similarly, no significant associations were found between group status (CLP and control) and the following categorical variables: maternal gingivitis, periodontitis, caries, gestational hypertension, gestational diabetes, urinary infection, antibiotic usage, or infant sex (all *p*-value > 0.05), suggesting these factors were independent of the lip and palate condition (Table 2).

**Table 2:** Statistical comparisons of the characteristics of study participants.

**Variable**	**Statistical test**	***p*-value**	**Effect size**
Maternal age in years	Wilcoxon rank sum test	0.721	Rank biserial = −0.05; 95% CI [−0.32–0.22]; CLP <Control
Gingivitis	Pearson’s Chi-squared test	0.1049	Odds ratio = 0.35; 95% CI [0.11–1.06]
Periodontitis	Fisher’s Exact Test	1	Odds ratio = 1.55; 95% CI [0.24–9.91]
Caries	Pearson’s Chi-squared test	0.1922	Odds ratio = 2.45; 95% CI [0.79–7.60]
Smoking during pregnancy	Fisher’s Exact Test	0.01026^*^	Odds ratio^A^ = 16.65; 95% CI [0.90–308.04]
Gestational hypertension	Pearson’s Chi-squared test	0.1958	Odds ratio = 0.39; 95% CI [0.12–1.28]
Gestational diabetes	Fisher’s Exact Test	0.3498	Odds ratio = 3.39; 95% CI [0.33–34.27]
Urinary infection	Pearson’s Chi-squared test	0.7909	Odds ratio = 0.78; 95% CI [0.30–2.03]
Antibiotic usage	Pearson’s Chi-squared test	0.09741	Odds ratio = 2.56; 95% CI [0.96–6.80]
**Children’s characteristics**			
Sex	Pearson’s Chi-squared test	0.1629	Odds ratio = 2.46; 95% CI [0.84–7.25]
Weeks of gestation	Wilcoxon rank sum test	0.05749	Rank biserial = −0.28; 95% CI [−0.52–0.01] CLP <Control
Child’s age during maternal sampling in days	Wilcoxon rank sum test	6.95e−10^*^	Rank biserial = 0.86; 95% CI [0.77–0.92]; CLP > Control

**Notes.**

95% CI Confidence Interval of 95%

* *p*-value < 0.05.

A to deal with the denominator equaling zero, 0.5 was added in all cells of the contingency table.

Two exceptions were noted. First, the child’s age at the time of maternal sample collection was significantly higher in the CLP group (*p*-value = 6.95E−10; Table 2; Fig. S1C). Second, smoking during pregnancy was significantly associated with the CLP group (*p*-value 0.010). Six participants in the CLP group reported smoking, compared to none in the control group. Greater similarity and homogeneity among participants reduced the potential influence of confounding variables, allowing a clearer signal from the variable of interest. This was the case for nearly all characteristics in this study, except the child’s age and smoking habit.

### Sequencing data

We obtained approximately 5.64 Gb of sequencing data, comprising 11,201,446 read pairs with an average length of 250 bp ± 28 bp. An average of 160,021 ± 99,918 read pairs per sample was obtained (Table S3). After quality filtering and processing with DADA2, 7,114,480 reads were assigned to 5,107 amplicon sequence variants (ASVs). Rarefaction curves based on ASV counts (Fig. S2 and Table S4) indicate that the sequencing depth captured most of the microbial diversity in all samples.

### Overview of diversity and community composition of the maternal oral microbiome

**Figure 1:**
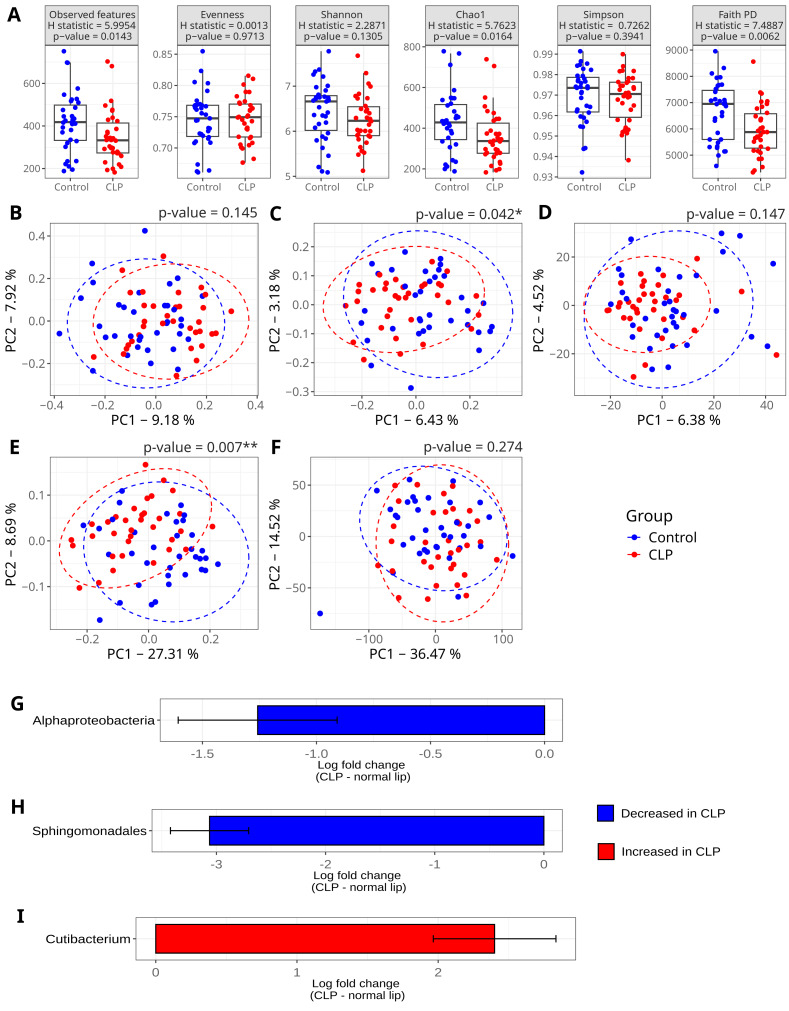
Microbial diversity comparisons between the CLP and control groups. Oral microbiome profiles of mothers of infants with cleft lip and palate (CLP group) and mothers of infants with a normal lip and palate (control group) were compared. Each dot represents one sample (participant) of the study. (A) Boxplots showing alpha diversity metrics. Statistical comparison was performed using the Kruskal-Wallis rank-sum test. (B-F) Principal Coordinate Analysis (PCoA) plots based on the following beta diversity distances: (B) Bray-Curtis, (C) Jaccard, (D) Aitchison, (E) Unweighted UniFrac, and (F) Weighted UniFrac. Figures from (G) to (I) refer to differential abundance analysis using ANCOM-BC, displaying only taxa with significantly different abundances (adjusted *p*-value < 0.05, Benjamini–Hochberg correction). The following taxonomic levels are shown: (G) class, (H) order, and (I) genus. The *x*-axis shows the log fold change between groups, with the control group as the reference.

Overall, the oral microbial diversity of mothers in the CLP and control groups showed broad similarities when considering taxa abundance, though significant differences were identified in the number of distinct taxonomic groups (detailed in the following section). Alpha diversity metrics using the Kruskal–Wallis sum rank test showed a similar distribution of Pielou’s evenness, Shannon, and Simpson metrics between the CLP and control groups (*p*-value > 0.05; Fig. 1A).

Beta diversity metrics reinforced this pattern of overall similarity between the CLP and control groups. Principal Coordinate Analysis (PCoA) plots showed substantial overlap between groups (Figs. 1B–1F). Permutational multivariate analysis of variance (PERMANOVA) found no significant separation (*p*-value > 0.05) based on Aitchison distance (accounting for compositionality), Bray-Curtis distance (abundance-based), or weighted Unifrac distance (phylogenetic and abundance-based) (all *p*-values > 0.05; Table 3).

At the phylum level, the community was dominated by Bacteroidota (formerly Bacteroidetes) (mean average in CLP: 28.3% ± 6.6%; control: 25.3% ± 7.7%), Pseudomonadota (formerly Proteobacteria) (CLP: 22.0% ± 8.5%; control: 21.7% ± 10.6%), and Bacillota (formerly Firmicutes) (CLP: 10.1% ± 8.2%; control: 10.7% ± 7.8%) (Fig. S3).

Bacteroidia was the most abundant class, comprising about one-quarter of the microbial composition (CLP: 28.3% ± 6.6%; control: 25.3% ± 7.7%), followed by Gammaproteobacteria (CLP: 22.0% ± 8.5%; control: 21.8% ± 10.6%) and Bacilli (CLP: 17.8% ± 8.0%; control: 18.1% ± 6.9%) (Fig. S4). The main representative order of Bacteroidia, Bacteroidales, correspondingly had the highest mean relative abundance among all orders (CLP: 27.6% ± 6.6%; control: 23.9% ± 7.9%) (Fig. S5). The most abundant families were *Bacteroidaceae*, *Pasteurellaceae*, *Streptococcaceae*, *Veillonellaceae*, *Neisseriaceae*, *Fusobacteriaceae*, *Porphyromonadaceae*, and *Micrococcaceae* (Fig. S6). The most abundant genera within each of these families were *Prevotella*, *Haemophilus*, *Streptococcus*, *Veillonella*, *Neisseria*, *Fusobacterium*, *Porphyromonas*, and *Rothia*, respectively (Fig. S7 and Table S5).

**Table 3:** PERMANOVA (adonis2 R package) of beta diversities between a group of mothers who had a child with a cleft lip and palate and a group of mothers who had a child with a normal lip and palate.

**All samples**					
Formula used = (Child’s sex + Urinary infection + Child age in days + Gingivitis + Caries + Gestational hypertension + Antibiotic usage) + Child lip and palate					
**Metric**	**SumOfSqs**	**R^2^**	**Residual R^2^**	**F**	***P*-value**
Aitchison	4,199	0.01891	0.85503	1.1058	0.147
Bray-Curtis	0.2622	0.02035	0.83080	1.2001	0.145
Jaccard	0.3547	0.02044	0.84898	1.1796	0.042^*^
Unweighted Unifrac	0.12442	0.04230	0.81162	2.5537	0.007^**^
Weighted Unifrac	7,542	0.02059	0.82940	1.2167	0.274

**Notes.**

* *p*-value < 0.05.

** *p*-value < 0.01.

999 permutations were used for the tests. See Table S7 for stratified comparisons of beta diversity metrics.

### Stratified analysis

We repeated diversity comparisons within subsets defined by clinical covariates. Alpha diversity remained similar between the CLP and control groups (*p*-value > 0.05) in most subsets: mothers of female infants; participants without gingivitis; participants with gingivitis; hypertensive participants; participants with antibiotic use; and participants with urinary infection (Table S6). Exceptions were found in participants with caries and those without antibiotic use, where Faith’s Phylogenetic Diversity differed significantly (*p*-value < 0.05, Table S6).

Similarly, beta diversity was not significantly different (*p*-value > 0.05) in the following stratified subsets: mothers of female infants; participants with healthy gingiva; participants with caries; non-hypertensive participants; hypertensive participants; and participants with a urinary infection (Table S7).

Functional prediction analysis using PICRUSt2 showed no significant differences in predicted metagenomic content (KEGG Orthology or Enzyme Commission numbers) between the CLP and control groups (*p*-value > 0.05).

### Despite overall similarity, significant differences exist between the oral microbiome of mothers in the CLP group and the oral microbiome of mothers in the control group

Although general similarities between the CLP and control groups were noted in the previous subsection, we identified some significant differences.

**Diversity**. Three alpha diversity metrics—Observed ASVs (observed features), Chao1, and Faith’s Phylogenetic Diversity—were significantly higher in the control group (respectively, *p*-value = 0.0143, 0.0164, and 0.0062) (Fig. 1A). Beta diversity also differed significantly between groups based on ASV presence/absence as measured by Jaccard distances (PERMANOVA *R*^2^ = 0.0204 *p*-value = 0.042) and unweighted Unifrac (PERMANOVA *R*^2^ = 0.0423, *p*-value = 0.007). This last diversity metric considers the phylogenetic distance of sequences. The first two principal coordinates (PC1 and PC2) in the Jaccard and unweighted Unifrac distance matrix accounted for about 9% and 36% of the total variability, respectively (Figs. 1C and 1E). These results indicate that while the overall abundance structure of communities was similar, differences were observed when considering the presence/absence of ASVs and the phylogenetic composition.

**Differential abundance**. ANCOM-BC revealed specific taxa driving these differences in microbial composition (Table S8). The order Sphingomonadales and its class, Alphaproteobacteria, were significantly depleted in the CLP group (72% and 95% reduction *versus* control, respectively; adjusted *p*-value < 0.05; Figs. 1G–1H and Table 4). In contrast, the genus *Cutibacterium* was over 11-fold more abundant in the CLP group (adjusted *p*-value < 0.05; Fig. 1I and Table 4).

**Table 4:** Changes in the taxa abundances of maternal oral microbiota.

**Taxonomy**	**Comparison**	**LFC**	**SE**	**Percent ∣ Fold change**	***p*-value**	**q-value**
Class Alphaproteobacteria	CLP *vs* control	−1.25778	0.3484	≈72% decrease	1.86e−3	0.0298
Order Sphingomonadales	CLP *vs* control	−3.062864	0.3582	≈95.3% decrease	6.25e−7	1.94e−5
Genus *Cutibacterium*	CLP *vs* control	2.399388	0.4342	≈11-fold higher	4.61e−5	0.0022
Phylum Cyanobacteriota	Only male CLP *vs* only male control	−2.778532	0.4982	≈93.8% decrease	1.66e−4	0.0022
Class Cyanobacteriia	Only male CLP *vs* only male control	−2.778532	0.4909	≈93.3% decrease	1.47e−4	0.0023
Order Cyanobacteriales	Only male CLP *vs* only male control	−2.778532	0.4937	≈93.3% decrease	1.54e−4	0.0030
Family *Coleofasciculaceae*	Only male CLP *vs* only male control	−3.326493	0.5116	≈96.4% decrease	6.87e−5	0.0034
Genus *Caldora*	Only male CLP *vs* only male control	−3.326493	0.4810	≈96.4% decrease	4.11e−5	0.0036
Family *Lactobacillaceae*	Only male CLP *vs* only male control	−1.643088	0.4473	≈80.7% decrease	3.67e−3	0.0458
Genus *Limosilactobacillus*	Only male CLP *vs* only male control	−2.699607	0.3113	≈93.3% decrease	3.22e−3	0.0355
Order Sphingomonadales	Without caries CLP *vs* without caries control	−3.895423	0.4918	≈97.9% decrease	1.28e−5	0.0004
Genus *Cutibacterium*	Without caries CLP *vs* without caries control	3.762912	0.4849	≈43-fold increase	8.71e−6	0.0008
Class Alphaproteobacteria	Non-hypertensive CLP *vs* non-hypertensive control	−1.823441	0.4168	≈83.9% decrease	0.0007	0.0120
Order Sphingomonadales	Non-hypertensive CLP *vs* non-hypertensive control	−3.457618	0.4174	≈96.9% decrease	3.39e−5	0.0011
Genus *Cutibacterium*	Non-hypertensive CLP *vs* non-hypertensive control	3.021573	0.5029	≈20.5-fold increase	1.30e−4	0.0120

**Notes.**

LFC Log Fold Change SE Standard Error *q*-value Benjamini–Hochberg adjusted *p*-value

Fold change was calculated as eˆ(LFC). Percent change was calculated as 1-eˆ(LFC). All taxa shown in the table passed the sensitivity test of ANCOM-BC2 (“passed_ss”).

**Figure 2:**
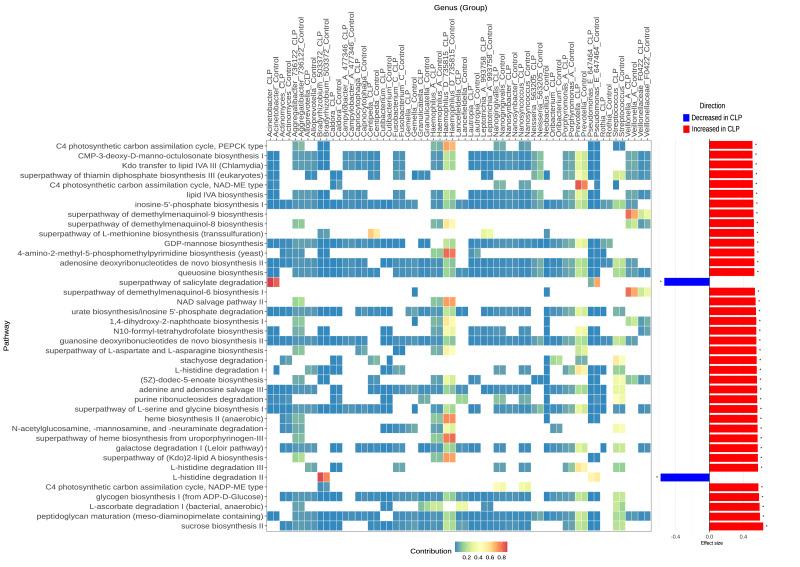
Predicted MetaCyc pathways differentially enriched in the CLP group compared to the control group. CLP = mothers of an infant with a cleft lip and palate. Control = mothers of an infant with a normal lip and palate. Shown are the 40 MetaCyc pathways with the largest effect sizes (bar plots representing median centered-log ratio (CLR) difference between groups divided by the maximum within-group CLR difference). Pathway abundances were predicted using PICRUSt2, and differential abundance was assessed with ALDEx2. Taxonomic contributions to each pathway (heatmap) represent the proportion of a given pathway’s predicted abundance attributed to a specific taxon in each sample.

**Predicted functional pathways**. Functional prediction (PICRUSt2/Metacyc database/ALDEx2) identified 158 differentially abundant pathways (151 enriched and seven depleted in the CLP group; adjusted *p*-value < 0.05; Tables S9 and S10). The 40 MetaCyc pathways with the largest effect sizes (Fig. 2) were related to five broad functional themes: (1) carbohydrate acquisition, storage, and degradation, (2) Gram-negative cell envelope/Lipopolysaccharide (LPS) potential, (3) anaerobe-associated cofactors, (4) host-glycan foraging, and (5) nucleotide biosynthesis and salvage, indicative of growth and replication capacity (Fig. 2 and Table S10), described in more details in the following numbered paragraphs. (1) Carbohydrate acquisition, storage, and degradation: Enriched pathways for carbohydrate acquisition and storage included Sucrose biosynthesis II (PWY-7238) and glycogen biosynthesis I (GLYCOGENSYN-PWY), with *Haemophilus_D*, *Prevotella*, and *Veillonella_A* contributing the most for both potential pathways (Fig. 2 and Table S10). Considering the carbohydrate degradation, galactose degradation I (PWY-6317) and stachyose degradation (PWY-6527) were potentially upregulated in the CLP group. Similar to carbohydrate anabolism (acquisition and storage), *Haemophilus_D*, *Prevotella*, and *Veillonella_A* also contributed the most to the predicted galactose degradation I. In contrast, *Oribacterium* and *Streptococcus* contributed the most to the predicted stachyose degradation. (2) Regarding Gram-negative cell envelope and LPS potential, we observed the following potential pathways enriched in the CLP group: peptidoglycan maturation involving meso-diaminopimelate (PWY0-1586), superpathway of (Kdo)2-lipid A biosynthesis (KDO-NAGLIPASYN-PWY), lipid IVA biosynthesis (NAGLIPASYN-PWY), Kdo transfer to lipid IVA III (PWY-6467), and CMP-3-deoxy-D-manno-octulosonate biosynthesis I (PWY-1269, CMP-KDO biosynthesis) (Fig. 2 and Table S10). Although this last pathway was categorized as Carbohydrate Biosynthesis, CMP-KDO is a precursor to the LPS biosynthesis. KDO is a carbohydrate that has a biological function of producing an activated nucleotide-sugar precursor necessary for assembling the LPS inner core in Gram-negative bacteria. All these differentially enriched pathways indicates a shift toward a more Gram-negative oral microbiome and an active cell-wall remodeling. Taxa that contributed the most to these pathways included *Haemophilus_* D, *Prevotella*, and *Veillonella_* A. *Aggregatibacter* had one of the highest contributions to the superpathway of (Kdo)2-lipid A biosynthesis. (3) For anaerobe-associated cofactors, pathways enriched in the CLP group included the superpathway of heme biosynthesis from uroporphyrinogen-III (PWY0-1415), heme biosynthesis II (anaerobic) (HEMESYN2-PWY), superpathway of demethylmenaquinol-6, -8 and -9 biosynthesis, and 1,4-dihydroxy-2-naphthoate biosynthesis I (PWY-5837) (Table S10). Functions of heme biosynthesis had the highest taxonomic contribution of *Haemophilus* _D*, Haemophilus* _A, and *Aggregatibacter,* while the superpathway of demethylmenaquinol-6 and -9 biosynthesis had the highest contribution of *Veillonella* _A and a genus classified by Greengenes2 as F0422 (*Veillonellaceae)*. 1,4-dihydroxy-2-naphthoate biosynthesis I is a key intermediate for menaquinone used in anaerobic respiration/electron transport. This pathway had the taxonomic contribution of *Aggregatibacter*, *Haemophilus* _D, *Haemophilus* _A, and *Veillonella* _A, similar to the superpathway of demethylmenaquinol-8 biosynthesis (Fig. 2). (4) We also highlight the superpathway of N-acetylglucosamine, N-acetylmannosamine, and N-acetylneuraminate degradation (GLCMANNANAUT-PWY) that was enriched in the CLP group, with the highest contribution of *Streptococcus*, *Haemophilus*, and *Aggregatibacter*. This pathway is related to the glycan and sialic acid degradation. Glycan is a complex carbohydrate that often contains amino sugars like N-acetylglucosamine or N-acetylglucosamine. (5) Pathways for nucleotide biosynthesis/salvage and urate/purine turnover included purine ribonucleosides degradation (PWY0-1296), adenine and adenosine salvage III (PWY-6609), guanosine deoxyribonucleotides *de novo* biosynthesis II (PWY-7222), urate biosynthesis/inosine 5′-phosphate degradation (PWY-5695), adenosine deoxyribonucleotides *de novo* biosynthesis II (PWY-7220), and inosine-5′-phosphate biosynthesis I (PWY-6123) (Table S10). Most of these pathways had the highest taxonomic contribution of *Haemophilus* _D, *Prevotella*, and *Streptococcus*. The only exception was purine ribonucleosides degradation that had the highest contribution of *Lancefieldella*, *Streptococcus*, and *Nanogingivalis*. Enrichment of nucleotide salvage and DNA precursor biosynthesis can indicate high replication rates and efficient reuse of nucleosides—often seen in biofilm with abundant extracellular DNA and high turnover.

Within the nucleotide biosynthesis, we highlight the pathway N10-formyl-THF biosynthesis (1CMET2-PWY) which is related to one-carbon (folate-related) pathway and supports nucleotide biosynthesis and methylation, processes required for microbial growth and nucleic-acid production. This pathway was enriched in the CLP group, indicating a community with enhanced biosynthetic readiness for growth and nucleic-acid turnover. Taxonomies that most contributed to this pathway were *Haemophilus*_D, *Prevotella*, and *Aggregatibacter*.

The differentially abundant genus *Cutibacterium*, enriched in the CLP group, had some contribution to potential pathways related to nucleotide biosynthesis and turnover, carbohydrate processing (sucrose biosynthesis I, glycogen biosynthesis, and galactose degradation I (Leloir pathway), L-ascorbate degradation I, superpathway of L-serine and glycine biosynthesis I, and L-histidine degradation I.

### Stratified analysis

Significant differences in diversity metrics were found in several subsets (mothers without urinary infection, mothers with gingivitis, and antibiotic use) (adjusted *p*-value < 0.05; Tables S6 and S7), but none yielded differentially abundant taxa (Table S8), possibly due to limited sample sizes. Subsets that met criteria for further differential abundance analysis (mothers without caries, non-hypertensive mothers, and mothers of a male newborn) presented similar differentially abundant taxa as the ones found in the unstratified data (Table S8), except for mothers of male infants, where more prominent differences were found (detailed in the next section).

### Significant differences between CLP and control groups in mothers of male infants

**Figure 3:**
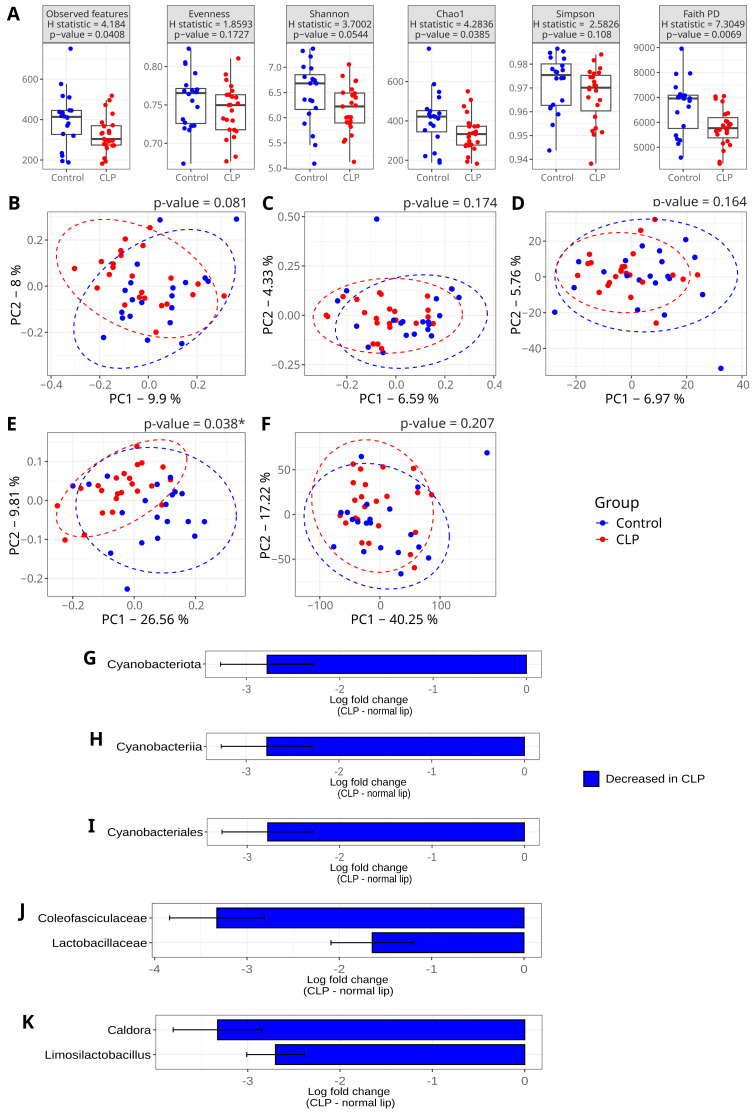
Diversity comparisons of the microbiome of mothers with a male child. CLP = oral microbiome of mothers who had a male child with CL/P. Control = oral microbiome of mothers who had a male child with a normal lip and palate. Each dot refers to a sample (participant) of the study. Boxplots in Figure (A) refer to alpha diversity metrics. The Kruskal–Wallis rank sum test was used to compare the alpha diversity metrics. Figures from B to F refer to Principal Coordinate Analysis (PCoA) using the following beta diversity distances: (B) Bray-Curtis, (C) Jaccard, (D) Aitchison, (E) Unweighted Unifrac and (F) Weighted Unifrac. Figures from (G) to (K) refer to differential abundance analysis using ANCOM-BC, displaying only taxa with significantly different abundances (adjusted *p* < 0.05, Benjamini–Hochberg correction). The following taxonomic levels are shown: (G) phylum, (H) class, (I) order, (J) family, and (K) genus. The *x*-axis shows the log fold change between groups, with the control group as the reference.

We found significant differences in alpha diversity metrics for the stratified analysis of mothers of a male infant. In this subset, alpha diversity metrics (Observed ASVs, Chao1, and Faith’s Phylogenetic Diversity) were significantly higher in the control group (*p* < 0.05; Fig. 3A). Beta diversity also differed significantly between groups based on the unweighted UniFrac distance, indicating that microbial composition is different between groups in terms of presence/absence of ASVs when taking into account the phylogenetic distance.

However, diversity metrics alone have limited interpretability without biological context (Shade, 2017). Alpha and beta diversities are general ways to identify differences between microbiomes, but they may not be sufficient to serve as biomarkers. Therefore, differential abundance analysis was performed to further untangle the diversity of the oral microbiome in this subset and investigate which taxa may contribute to the differences found in alpha and beta diversity of mothers of a male infant.

The phylum Cyanobacteriota was significantly depleted in the CLP group (93.8% decrease) (Fig. 3 and Table 4). Corresponding taxa at lower taxonomic levels—including the class Cyanobacteria, order Cyanobacteriales, family *Coleofaciculaceae*, and genus *Caldora* (96.4% decrease)—were also depleted in the CLP group (Table 4) (but see further results below). Furthermore, the family *Lactobacillaceae* and the genus *Limosilactobacillus* (93.3% decrease) were also differentially reduced in the CLP group (Fig. 3).

Functional prediction (PICRUSt2/Metacyc database) for this stratum identified 169 MetaCyc metabolic pathways significantly enriched in the CLP group (adjusted *p*-value < 0.05; Tables S11 and S12) and seven depleted. The most abundant enriched categories were “Cofactor, Prosthetic Group, Electron Carrier, and Vitamin Biosynthesis”, followed by “Amino Acid Biosynthesis” and “Nucleoside and Nucleotide Biosynthesis” (Table S11). Similar to the unstratifed data, all seven depleted pathways belonged to the Degradation/Utilization/Assimilation category, including five for aromatic compound degradation. While five aromatic compound degradation pathways were depleted in the CLP group, no pathways for the same category was enriched in the CLP group.

**Figure 4:**
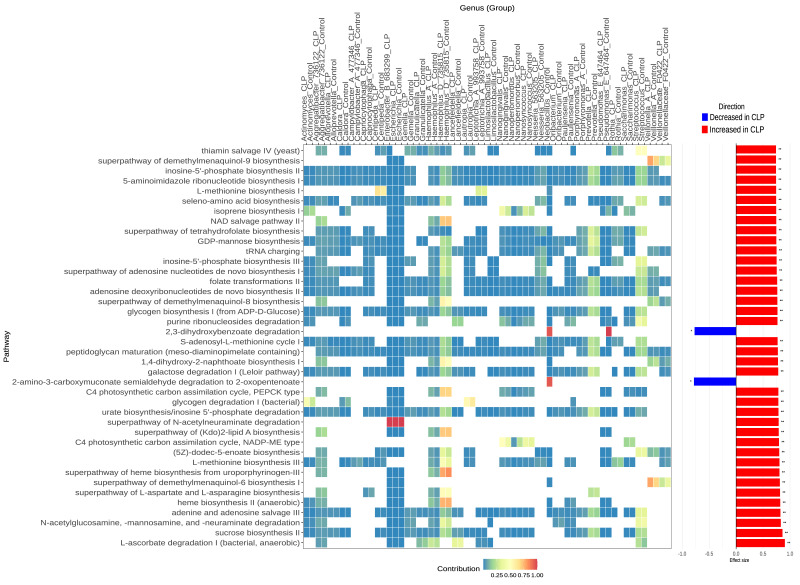
Predicted MetaCyc pathways differentially enriched in the CLP group compared to the control group stratified for mothers of male infants. CLP = mothers of a male infant with a cleft lip and palate. Control = mothers of a male infant with a normal lip and palate. Shown are the 40 MetaCyc pathways with the largest effect sizes (barplots representing median centered-log ratio (CLR) difference between groups divided by the maximum within-group CLR difference). Pathway abundances were predicted using PICRUSt2, and differential abundance was assessed with ALDEx2. Taxonomic contributions to each pathway (heatmap) were calculated using the pathway_pipeline.py script from PICRUSt2, representing the proportion of a given pathway’s predicted abundance attributed to a specific taxon in each sample. The red and blue bar plots on the right show the effect sizes of MetaCyc pathways.

The 40 pathways with the largest effect sizes (*p*-value < 0.05) (Fig. 4) in this stratum reinforced themes of Gram-negative metabolism (LPS), anaerobic respiration/cofactor biosynthesis (heme, menaquinones), and nucleotide turnover, with the similar taxonomic contribution as in unstratified data.

Notably, L-ascorbate degradation I (PWY0-301), related to anaerobic carbon catabolism, had the greatest effect size among pathways enriched in the CLP group (Fig. 4 and Table S12). We also observed an expansion of enriched pathways related to folate and methionine metabolism, comprising folate transformations II (PWY-3841), superpathway of tetrahydrofolate biosynthesis (PWY-6612), L-methionine biosynthesis III (HSERMETANA-PWY), L-methionine biosynthesis I (HOMOSER-METSYN-PWY), and S-adenosyl-L-methionine cycle I (PWY-6151). These pathways, crucial for nucleotide biosynthesis and methylation, were primarily driven by *Haemophilus*_D, *Prevotella*, and *Streptococcus*, suggesting enhanced biosynthetic activity and growth potential in the CLP-associated microbiome of mothers carrying male fetuses.

### Additional verification of taxonomic classifications

As described in Methods, we used the script greengenes2 non-v4-16s and the GreenGenes2 database to obtain taxonomic classifications. Because our results show that certain taxa (*Cutibacterium*, *Limosilactobacillus*, and *Caldora*) were differentially represented, we decided to perform additional verifications only for these taxa using BLASTn against the GenBank database core-nt. This additional step confirmed the *Cutibacterium* and *Limosilactobacillus* classifications (meaning that there were several top hits with 100% identity belonging to these taxa). However, for ASVs assigned to *Caldora*, we observed only 84% identity. On the other hand, there were many top hits with at least 99% identity to sequences classified as “Uncultured bacterium”, from a variety of sources. Some of these sources are related to the oral microbiome. For example, for sequence LC355487, isolation source is given as “sputum”; for sequence KM277709, isolation source is given as “saliva from wild Tibetan macaque”. We conclude that ASVs classified as *Caldora* most likely belong not to *Caldora* but to an as-yet unnamed genus, probably belonging to class Cyanophyceae (to which *Caldora* belongs).

## Discussion

Non-syndromic cleft lip with or without palate (NS-CL/P) can be caused by a combination of genetic and environmental factors (Dixon et al., 2011; Nasreddine, El Hajj & Ghassibe-Sabbagh, 2021). Our analysis revealed significant differences in the oral microbiome between mothers of infants with NS-CL/P and control mothers. These differences were more pronounced when the data was subset for mothers of male infants.

Before interpreting these differences, an important methodological consideration must be noted: all samples were collected *postpartum*, as the diagnosis of CL/P is made after birth, in most regions of Brazil. Access to treatment for neonates with CL/P within the first month of life is limited in Brazilian hospitals, making it challenging to obtain samples from mothers during pregnancy or the immediate *postpartum* period. Nevertheless, the differences we observed provide valuable clues that may help in understanding the role of the maternal oral microbiome in the development of the CL/P condition.

One key finding was the significantly increased abundance of the genus *Cutibacterium* in the CLP group compared to the control, as determined by ANCOM-BC. Species of this genus comprise Gram-positive, anaerobic (often described as aerotolerant anaerobic/facultatively anaerobic) bacteria that were historically classified as *Propiobacterium* and subsequently reclassified (Scholz & Kilian, 2016). Species of this genus are usually associated with human skin (Corvec, 2018), but they were also reported to colonize several human organs, such as the oral cavity and the gut (Scholz & Kilian, 2016). Because *Cutibacterium*, mainly *Cutibacterium acnes*, is usually described as a component of human-associated microbiota, this genus can be interpreted as a commensal member that can take the role of an opportunistic pathogen. Two strains of *Cutibacterium acnes* and one of *Cutibacterium namnetense* were isolated from endodontic lesions, indicating the possibility of opportunistic infections in the root canal during oral health care procedures (Álvarez-Muñoz et al., 2025).

We speculate that when the oral microbiota is in dysbiosis, *Cutibacterium* can influence maternal levels of pro-inflammatory molecules, which can induce, for example, the epigenetic silencing of the cell adhesion protein E-cadherin (CDH1) gene in embryonic cells. E-cadherin has been highlighted as a key epithelial adhesion regulator whose disruption can contribute substantially to NS-CL/P (Vogelaar et al., 2013; Cox et al., 2018). The inhibition of CDH1 gene further reduces E-cadherin levels, severely impairing the ability of cells to migrate and form the face properly, leading to NS-CL/P. This speculation is supported by the exposure of embryonic cells (neural crest cells) to pro-inflammatory molecules that induced methylation of the CDH1 promoter, which has been associated with the occurrence of oral cleft in mice (Alvizi et al., 2017; Alvizi et al., 2023).

Moreover, *Cutibacterium acnes*, generally studied due to its association with acne, can induce a pro-inflammatory response. Its cell wall components (peptidoglycan and lipoteichoic acid) can be recognized by binding toll-like receptors (TLRs) on the surface of host’s macrophages and monocytes, activating the TLR signaling pathway and triggering important intracellular signaling pathways. This causes nuclear translocation of transcription factors, which leads to the production of several pro-inflammatory cytokines including interleukin (IL) 1β, IL-8, and IL-6 and tumor necrosis factor (TNF) α (Ryu et al., 2015; Nguyen et al., 2018; Nguyen & Kim, 2020). Although the inflammatory response caused by *C. acnes* is usually associated with skin disease, the cell wall components of the *Cutibacterium* genus may increase maternal levels of pro-inflammatory molecules, contributing to placental inflammation (Challier et al., 2008; Couture et al., 2023). This may lead to the inhibition of CDH1 gene expression, compromising the epithelial adhesion and leading to the incomplete tissue fusion during fetal development.

Another way by which the increase in *Cutibacterium* could influence the CLP outcome during maternal gestation is by affecting the microbial structure in the oral habitat. Species of this genus can release fermentation products that can inhibit the growth of some microorganisms. They can also form biofilms that protect some members of the microbial community (Coenye, Spittaels & Achermann, 2021). The predicted sucrose and glycogen biosynthesis pathways observed in the PICRUSt2 results indicate that the microbial community is capable of creating a stable biofilm. These microbial interactions, in addition to metabolic, hormonal, nutritional, and immunological changes during pregnancy, can change the microbial community structure, deplete beneficial bacteria, increase susceptibility to oral diseases (Balan et al., 2018), and potentially contribute to adverse neonatal outcomes (Ye & Kapila, 2021; Xu & Han, 2022).

Furthermore, we observed potential pathways for Gram-negative bacteria (like Lipid A/LPS biosynthesis) enriched in the maternal oral microbiome of the CLP group. LPS, a component of Gram-negative cell walls, can trigger the systemic pro-inflammatory response in mothers, which can decrease the levels of E-cadherin and lead to the NS-CL/P outcome.

The pro-inflammatory hypothesis here proposed may have a disproportionate impact depending on fetal sex. This is relevant, as the prevalence of CL/P is about twice as high in males as in females (Shaw, Croen & Curry, 1991; Brito et al., 2011; Dixon et al., 2011; Barbosa Martelli et al., 2012). Maternal hormone levels and immunity responses can adapt differently to infection and inflammation in male and female fetuses. Male fetuses prioritize rapid growth (cell proliferation) and metabolism, potentially at the cost of a robust immune response. On the other hand, female fetuses invest more in placental energy reserves and immunity, as well as cytokine-mediated signaling. This balance between rapid growth and immune response to the environment may underlie the increased risk of adverse outcomes in male pregnancies (Clifton, 2005; Baines & West, 2023). Furthermore, fetal sex influences maternal endocrine and immune responses, which can subsequently alter the maternal oral microbiome during pregnancy, potentially explaining the differential outcomes observed between mothers carrying male and female fetuses (Ye & Kapila, 2021; Saadaoui, Singh & Al Khodor, 2021).

In our cohort, the genus *Limosilactobacillus* was significantly more abundant in control mothers of male infants than in mothers of male infants belonging to the CLP group, a difference not observed in mothers of female infants. *Limosilactobacillus* (previously classified as part of the genus *Lactobacillus*) is an anaerobic or aerotolerant Gram-positive bacterium. It comprises several probiotic bacteria, with *Limosilactobacillus reuteri* as one of the species known. We speculate that the decreased abundance of this genus in the CLP group compared with controls indicate that they are capable of maintaining a less dysbiotic oral community state. *Limosilactobacillus* has been investigated for its role as an oral probiotic capable of suppressing periodontal pathogens, and modulating an anti-inflammatory effect (Park et al., 2023; Boisen et al., 2023; Ananda, Suniarti & Bachtiar, 2024; Tarannum et al., 2024).

We briefly mention that we observed results similar to those of *Limosilactobacillus* for a group of ASVs that most likely belong the Cyanophyceae class. The fact that this taxon was observed in this study and that almost nothing is known about it points to the fact that there is still much to be discovered about the human oral microbiome.

The predicted upregulation of numerous metabolic pathways in mothers of male children with CLP indicates potential shifts in microbial metabolic priorities, such as energy utilization and signaling pathways (Pudlo et al., 2015; Romine et al., 2017). The categories with the most upregulated pathways were “Nucleoside and Nucleotide Biosynthesis”, “Cofactor, Prosthetic Group, Electron Carrier, and Vitamin Biosynthesis” and “Amino Acid Biosynthesis”, indicating a higher potential for microbial growth and replication in the CLP group. These pathways are involved in fundamental cellular functions and extracellular matrix constitution (Shames, Auweter & Finlay, 2009). We hypothesize that these pathways could be linked to maternal dietary habits, or may reflect a state of local dysbiosis associated with oral disease (Aleti et al., 2019).

Substantial evidence supports a bilateral relationship between oral and systemic diseases, where periodontal disease is a risk factor for systemic conditions, such as cardiovascular, renal, osteoarticular, respiratory, diabetes, oncological predisposition, stroke, and adverse pregnancy outcomes, among other clinically important medical conditions. The association occurs through mechanisms that may involve the dissemination of periodontal pathogens, their virulence factors, and local inflammatory mediators through bloodstream or the oral-intestinal axis (Hajishengallis & Chavakis, 2021; Meto et al., 2024).

We found no significant association between gingivitis and the NS-CL/P outcome. Although four beta diversity metrics (Aitchison, Bray-Curtis, Jaccard, and unweighted Unifrac) showed significant differences between the CLP and control groups of mothers with gingivitis, no differentially abundant taxa were observed. This may be due to the fact that most cases of gingivitis in our cohort were mild. While periodontitis is defined as irreversible bone resorption, gingivitis is prevalent in the human population and precedes reversible gum disease that can become periodontitis if unchecked. Mild gingival inflammation is considered a controlled state that can be prevented and does not contribute directly to dental tissue destruction (Lamont, Koo & Hajishengallis, 2018). According to the polymicrobial synergy and dysbiosis (PSD) hypothesis, environmental factors can select for a dysbiotic microbial community that disrupts the healthy microbiome and the host homeostasis (Hajishengallis & Lamont, 2012). Our ability to compare severe gingivitis or periodontitis with a healthy oral state was limited by the small number of such cases.

We opted for non-invasive assessments only, such as saliva collection and visual inspection of the gingival health of mothers between 0 and 30 days after giving birth. The absence of a more rigorous clinical assessment of periodontal health, such as full mouth periodontal examination, is a study limitation.

Future studies should aim to collect maternal samples closer to the time of delivery, include more cases of periodontitis, include the collection of maternal pro-inflammatory cytokines levels, and increase the overall sample size to better elucidate the relationship between the oral microbiome and NS-CL/P. Collecting samples during pregnancy would be valuable. Finally, mechanistic studies are needed to delineate the potential role of the oral microbiome as an epigenetic factor in NS-CL/P.

## Conclusions

In sum, while the overall oral microbiome structure was largely similar between mothers of neonates with NS-CL/P and controls, differences were identified, in particular the higher abundance of *Cutibacterium* in the CLP group. The stratified subset of mothers of male infants with CLP revealed a distinct microbial richness and, most notably, a significant depletion of *Limosilactobacillus* and a potential enrichment of numerous metabolic pathways. These findings suggest that the maternal oral microbiome, particularly in the context of male offspring, may point to an interaction between maternal oral microbiota and the NS-CL/P risk. As far as the authors could determine, this is the first study that provides molecular evidence about this link.

In terms of social impact, our results suggest the need to promote improved better health programs, including maternal oral hygiene before and during pregnancy, particularly among low-income populations. If the hypotheses raised in this work are correct, such actions should lead to a decrease in the incidence of NS-CL/P among the most vulnerable groups.

### Limitations

• All samples were collected after birth. Nine CLP samples were sampled beyond 30 days after birth. 33 samples of control and two samples of CLP were collected within three days after birth.
• 16S rRNA can be used for reliable taxonomic identification down to the genus level, but not down to the species level.
• Functional predictions were based on the 16S rRNA data. Total DNA and transcriptomic data would be required to explore functions with more certainty.
• Only saliva was collected due to the non-invasiveness of the assessment. Other parts of the oral cavity such as subgingival plaque were not sampled.
• Including dietary data, a wider range of periodontitis cases, and pro-inflammatory levels of cytokines could enrich the study.

##  Supplemental Information

10.7717/peerj.21128/supp-1 Supplemental Information 1 Histogram of characteristics of study participants CLP = group of mothers with a baby with cleft lip or palate. Control = group of mothers with a baby with normal lip and palate. A) Age of mothers. B) Weeks of gestation. C) Children’s age in days. D) Modified Gingival Index (MGI) based on five categories, 0-4 scale, that score the marginal and papillary gingival tissue based on color, texture, edema, and spontaneous bleeding (Lobene et al., 1986).

10.7717/peerj.21128/supp-2 Supplemental Information 2 Rarefaction curves for observed ASVs of samples from the oral microbiome of mothers who had babies with cleft lip and palate (CLP group) and mothers with babies with normal lip and palate (control) R Vegan package was used with a step size of 4,000 counts from the ASV table to generate the rarefaction curves. The X-axis refers to the number of randomly sampled reads without replacement and the y-axis to the number of estimated ASVs for the corresponding number of sampled reads. The number at the end of each curve refers to a Sample ID. Red lines are samples from the CLP group and blue lines are samples from the control.

10.7717/peerj.21128/supp-3 Supplemental Information 3 Relative abundance in phylum level of the oral microbiome of mothers who had a child with cleft lip or palate (CLP) and mothers who had a child with normal lip and palate (control) The x-axis refers to sample IDs followed by the child’s age in days during maternal sampling.

10.7717/peerj.21128/supp-4 Supplemental Information 4 Relative abundance in class level of the oral microbiome of mothers who had a child with cleft lip or palate (CLP) and mothers who had a child with normal lip and palate (control) The x-axis refers to sample IDs followed by the child’s age in days during maternal sampling.

10.7717/peerj.21128/supp-5 Supplemental Information 5 Relative abundance in order level of the oral microbiome of mothers who had a child with cleft lip or palate (CLP) and mothers who had a child with normal lip and palate (control) The x-axis refers to sample IDs followed by the child’s age in days during maternal sampling.

10.7717/peerj.21128/supp-6 Supplemental Information 6 Relative abundance in the family level of the oral microbiome of mothers who had a child with cleft lip or palate (CLP) and mothers who had a child with normal lip and palate (control) The x-axis refers to sample IDs followed by the child’s age in days during maternal sampling.

10.7717/peerj.21128/supp-7 Supplemental Information 7 Relative abundance in the genus level of the oral microbiome of mothers who had a child with cleft lip or palate (CLP) and mothers who had a child with normal lip and palate (control) The x-axis refers to sample IDs followed by the child’s age in days during maternal sampling.

10.7717/peerj.21128/supp-8 Supplemental Information 8 Questionnaire about health history and habits of the study participants

10.7717/peerj.21128/supp-9 Supplemental Information 9 Supplemental Tables

## Additional Information and Declarations

### Data Availability

The following information was supplied regarding data availability: The sequences are available at NCBI Sequence Read Archive: PRJNA1066742.
