# Supplementary material for: The oral maternal microbiome plays a role in the development of cleft lip and palate condition in children
Source: PeerJ. 2026 Apr 27;14:e21128. doi: 10.7717/peerj.21128 (PMC13131353; doi:10.7717/peerj.21128)
Supplement: Supplemental Information 8 [file peerj-14-21128-s008.docx]

**Supplementary Document**

**Document S1:** Questionnaire about health history and habits of the study participants

Level of education

⬚ Illiterate ⬚ Incomplete Tertiary Education

⬚ Incomplete Primary Education ⬚ Complete Tertiary Education

⬚ Complete Primary Education ⬚ Incomplete Post-graduate degree

⬚ Incomplete Secondary Education ⬚ Complete Post-graduate degree

⬚ Complete Secondary Education

Ethnicity

⬚ White ⬚ Indigenous

⬚ Brown ⬚ Asian

⬚ Black

Health questionnaire

Have you ever had or currently have any of the following illnesses?

⬚ Heart disease ⬚ Asthma/bronchitis

⬚ High blood pressure ⬚ Digestive disorders

⬚ Liver disease ⬚ Cancer

⬚ Kidney disease ⬚ Diabetes

⬚ Psychiatric disorder ⬚ Tuberculosis

⬚ Epilepsy/seizures ⬚ HIV virus

Do you get sick frequently? ⬚ Yes ⬚ No

Are you undergoing medical treatment and taking any medication?

⬚ Yes Which? _____________________________________ ⬚ No

When you are sick, do you always go to the doctor, or do you take medicine "alone" (self-medicate)?

⬚ Yes Which? _____________________________________ ⬚ No

Prenatal and birth information

Did you have any prenatal consultations during this pregnancy?

⬚ Yes; How many times? ______ ⬚ No

Which service(s) did you consult?

⬚ Sistema Único de Saúde (SUS) ⬚ Private appointment

⬚ Healthcare plan ⬚ I don't know

How many months were you pregnant when you had your first prenatal appointment? ______

Did you do any tests during this pregnancy?

⬚ Yes ⬚ No ⬚ I don’t know

⬚ Blood

⬚ Urine

Do you know if there were any abnormal findings?

⬚ Yes; Which? ____________________________________ ⬚ No ⬚ I don’t know

Was a test for syphilis and hepatitis carried out during prenatal care?

⬚ Yes ⬚ No

What were the results of the tests for syphilis and hepatitis?

⬚ Positive ⬚ Negative ⬚ I don’t know

Was testing for AIDS and toxoplasmosis offered during prenatal care?

⬚ Yes ⬚ No ⬚ I don’t know

What was the test result for AIDS and toxoplasmosis?

⬚ Positive ⬚ Negative ⬚ I don’t know

Did you have a fever during pregnancy?

⬚ Yes ⬚ No ⬚ I don’t know

Did you use antibiotics?

⬚ Yes; Which? ____________________________________ ⬚ No ⬚ I don’t know

Did you have anemia during pregnancy?

⬚ Yes ⬚ No ⬚ I don’t know

During pregnancy, did any healthcare professional prescribe iron and/or folic acid to prevent or treat anemia?

⬚ Yes, iron ⬚ No ⬚ I don’t know

⬚ Yes, folic acid

⬚ Yes, both

Did you use it? ⬚ Yes ⬚ No

Did you have complications during birth?

⬚ Yes ⬚ No ⬚ I don’t know

Gestational age category

⬚ Premature ⬚ Normal Weeks: ____________

Oral health assessment

Number of teeth present in the mouth = _____

Do you wear dentures? For how long?

⬚ Yes ⬚ Total, bimaxillary ⬚ Total, unimaxillary

⬚ Yes, removable ⬚ No

Do you brush your teeth? Frequency/day

⬚ Yes ⬚ Once a day ⬚ Twice a day ⬚ 3X a day

⬚ More than 3x a day ⬚ No

Do you use dental floss? Frequency/day

⬚ Yes ⬚ Once a day ⬚ Twice a day ⬚ 3X a day

⬚ More than 3x a day ⬚ No

Do you have a habit of brushing your tongue?

⬚ Yes, always ⬚ Yes, sometimes ⬚ No

When? ____________

Do your gums bleed?

⬚ Yes, always ⬚ Yes, sometimes ⬚ No

When? ____________

Do you notice your teeth moving?

⬚ Yes; How many months? _______________ ⬚ No

Are you a smoker?

⬚ Former smoker ⬚ Yes ⬚ No

Which habits do you have?

⬚ Bite nails ⬚ Grind/clench your teeth ⬚ Bite objects

Do you go to the dentist? Frequency/year

⬚ Yes ⬚ Once a year ⬚ Twice a year ⬚ More than twice a year

Have you ever had oral hygiene guidance?

⬚ Yes ⬚ No ⬚ I don’t know

Have you ever had periodontal treatment (gum treatment)? ⬚ Yes ⬚ No

During pregnancy, did you have any dental problems?

⬚ Yes; Which? __________________________ ⬚ No

Did you use antibiotics?

⬚ Yes; Which? __________________________ ⬚ No

Type of orofacial cleft in the child (for the group of mothers of children with NS-CL/P)

⬚ Field not filled 🡪 mother of the control Group

⬚ Unilateral cleft lip: ⬚ right ⬚ left

⬚ Bilateral cleft lip

⬚ Unilateral cleft lip and palate pre-incisive foramen: ⬚ right ⬚ left

⬚ Bilateral cleft lip and palate pre-incisive foramen

⬚ Unilateral cleft lip and palate with incisive foramen involvement: ⬚ right ⬚ left

⬚ Bilateral cleft lip and palate with incisive foramen involvement

⬚ Complete posterior cleft of the palate (soft and hard palate) with incisive foramen involvement

⬚ Incomplete posterior cleft of the palate: ⬚ soft palate) ⬚ hard palate"

⬚ Bifid uvula
