# Supplementary material for: The oral maternal microbiome plays a role in the development of cleft lip and palate condition in children
Source: PeerJ. 2026 Apr 27;14:e21128. doi: 10.7717/peerj.21128 (PMC13131353; doi:10.7717/peerj.21128)
Supplement: Supplemental Information 9 [file peerj-14-21128-s009.zip › peerj-117017-supplementary_tables_S1_to_S9_and_S11.docx]

**Supplementary Tables 1**

**Table S1:** Primers used for amplicon sequencing

| Primers | Sequence |
| --- | --- |
| 16S Amplicon Forward 5' | TCGTCGGCAGCGTCAGATGTGTATAAGAGACAGCCTACGGG  NGGCWGCAG |
| 16S Amplicon Reverse 5' | GTCTCGTGGGCTCGGAGATGTGTATAAGAGACAGGACTACH  VGGGTATCTAATCC |
| Forward overhang 5’ | TCGTCGGCAGCGTCAGATGTGTATAAGAGACAG |
| Reverse overhang 5' | GTCTCGTGGGCTCGGAGATGTGTATAAGAGACAG |

**Table S2:** Normality test using Shapiro-Wilk in metadata (characteristics of study participants)

| **Variables** | **W statistic** | **p-value** | **Normal distribution** |
| --- | --- | --- | --- |
| Maternal age | 0.9555 | 0.01633 | No |
| Weeks of gestation | 0.70252 | 8.029e-10 | No |
| Children's age during maternal sampling | 0.69277 | 1.49e-10 | No |

Null hypothesis of normal distribution was rejected if p-value < 0.05

**Table S3:** Sequencing data and accession list

| SampleID | Paired raw reads | Reverse raw reads | Sum of sequence length (forward) | Sum of sequence length (reverse) | Number of sequences after DADA2 |
| --- | --- | --- | --- | --- | --- |
| S01 | 178,293 | 178,293 | 44,718,613 | 44,708,457 | 115,100 |
| S02 | 153,635 | 153,635 | 38,537,871 | 38,535,729 | 98,569 |
| S03 | 110,371 | 110,371 | 27,592,130 | 27,605,457 | 71,439 |
| S04 | 101,206 | 101,206 | 25,379,342 | 25,377,214 | 67,007 |
| S05 | 86,208 | 86,208 | 21,616,522 | 21,615,791 | 51,047 |
| S06 | 77,853 | 77,853 | 19,526,229 | 19,526,563 | 47,846 |
| S07 | 92,901 | 92,901 | 23,300,400 | 23,298,900 | 59,533 |
| S08 | 127,299 | 127,299 | 31,927,858 | 31,919,652 | 80,425 |
| S10 | 159,642 | 159,642 | 40,032,998 | 40,034,352 | 97,142 |
| S11 | 130,019 | 130,019 | 32,614,964 | 32,596,796 | 86,652 |
| S12 | 88,472 | 88,472 | 22,183,510 | 22,181,681 | 56,090 |
| S13 | 88,485 | 88,485 | 22,151,874 | 22,159,099 | 58,814 |
| S14 | 74,636 | 74,636 | 18,673,107 | 18,673,262 | 50,066 |
| S15 | 180,473 | 180,473 | 45,257,828 | 45,247,375 | 119,165 |
| S16 | 150,441 | 150,441 | 37,727,172 | 37,720,764 | 99,055 |
| S17 | 177,159 | 177,159 | 44,436,319 | 44,433,364 | 112,931 |
| S18 | 170,999 | 170,999 | 42,891,806 | 42,865,788 | 104,074 |
| S19 | 73,289 | 73,289 | 18,373,477 | 18,372,690 | 45,981 |
| S21 | 125,828 | 125,828 | 31,550,097 | 31,551,556 | 80,943 |
| S22 | 202,691 | 202,691 | 50,832,801 | 50,832,398 | 131,902 |
| S23 | 172,321 | 172,321 | 43,203,979 | 43,187,880 | 118,483 |
| S24 | 145,417 | 145,417 | 36,441,295 | 36,431,330 | 90,008 |
| S25 | 87,641 | 87,641 | 21,973,889 | 21,971,700 | 54,735 |
| S26 | 62,341 | 62,341 | 15,637,416 | 15,631,786 | 37,129 |
| S27 | 67,357 | 67,357 | 16,883,950 | 16,876,097 | 40,658 |
| S29 | 117,378 | 117,378 | 29,432,599 | 29,434,168 | 73,020 |
| S30 | 179,300 | 179,300 | 44,965,083 | 44,959,224 | 118,994 |
| S31 | 128,880 | 128,880 | 32,306,226 | 32,310,737 | 87,275 |
| S32 | 147,216 | 147,216 | 36,886,194 | 36,874,880 | 100,545 |
| S33 | 139,174 | 139,174 | 34,848,380 | 34,848,029 | 88,609 |
| S34 | 154,458 | 154,458 | 38,745,338 | 38,742,747 | 108,837 |
| S35 | 191,573 | 191,573 | 48,047,524 | 48,041,029 | 111,097 |
| S36 | 277,172 | 277,172 | 69,498,611 | 69,490,551 | 160,739 |
| S37 | 94,559 | 94,559 | 23,714,968 | 23,715,650 | 59,549 |
| S38 | 87,975 | 87,975 | 22,069,368 | 22,066,101 | 64,441 |
| S39 | 107,170 | 107,170 | 26,873,648 | 26,872,474 | 67,649 |
| S40 | 150,372 | 150,372 | 37,666,407 | 37,666,908 | 85,375 |
| S41 | 111,299 | 111,299 | 27,894,544 | 27,900,234 | 68,492 |
| S42 | 139,438 | 139,438 | 34,944,188 | 34,947,914 | 90,565 |
| S43 | 178,195 | 178,195 | 44,681,063 | 44,675,329 | 100,372 |
| S44 | 113,193 | 113,193 | 28,367,617 | 28,381,511 | 71,296 |
| S45 | 63,969 | 63,969 | 16,040,893 | 16,038,486 | 37,444 |
| S46 | 59,938 | 59,938 | 15,027,298 | 15,031,098 | 38,681 |
| S47 | 256,772 | 256,772 | 64,339,524 | 64,338,158 | 165,402 |
| S48 | 158,641 | 158,641 | 39,725,409 | 39,740,249 | 101,119 |
| S49 | 174,817 | 174,817 | 43,776,637 | 43,777,470 | 106,640 |
| S50 | 223,691 | 223,691 | 56,070,511 | 56,095,356 | 140,731 |
| S51 | 150,624 | 150,624 | 37,747,610 | 37,752,842 | 96,651 |
| S52 | 160,767 | 160,767 | 40,324,341 | 40,320,356 | 107,360 |
| S53 | 46,981 | 46,981 | 11,776,954 | 11,775,592 | 27,243 |
| S54 | 223,674 | 223,674 | 56,060,167 | 56,044,209 | 143,158 |
| S55 | 168,907 | 168,907 | 42,316,585 | 42,313,112 | 104,729 |
| S56 | 90,350 | 90,350 | 22,643,097 | 22,651,241 | 54,062 |
| S57 | 126,493 | 126,493 | 31,693,203 | 31,691,638 | 84,021 |
| S58 | 184,673 | 184,673 | 46,278,127 | 46,287,480 | 124,258 |
| S59 | 71,205 | 71,205 | 17,851,007 | 17,849,899 | 44,492 |
| S60 | 106,156 | 106,156 | 26,599,044 | 26,597,124 | 74,330 |
| S61 | 118,732 | 118,732 | 29,771,434 | 29,766,798 | 72,204 |
| S62 | 140,154 | 140,154 | 35,160,766 | 35,160,260 | 100,003 |
| S63 | 85,422 | 85,422 | 21,394,814 | 21,391,845 | 52,956 |
| S64 | 157,578 | 157,578 | 39,505,074 | 39,513,385 | 105,379 |
| S65 | 139,926 | 139,926 | 35,084,683 | 35,074,487 | 82,106 |
| S66 | 348,901 | 348,901 | 87,400,093 | 87,426,899 | 232,868 |
| S67 | 574,615 | 574,615 | 144,067,090 | 144,023,046 | 330,974 |
| S68 | 71,479 | 71,479 | 17,894,400 | 17,895,091 | 49,886 |
| S69 | 464,980 | 464,980 | 116,616,492 | 116,595,000 | 285,438 |
| S70 | 496,119 | 496,119 | 124,288,808 | 124,328,609 | 327,918 |
| S71 | 252,811 | 252,811 | 63,200,537 | 63,204,507 | 159,999 |
| S72 | 388,585 | 388,585 | 97,471,709 | 97,435,315 | 248,163 |
| S73 | 292,157 | 292,157 | 73,177,218 | 73,190,330 | 184,686 |
| Total | 11,201,446 | 11,201,446 | 2,807,710,730 | 2,807,593,049 | 7,114,550 |
| Mean | 160,021 | 160,021 | 40,110,153 | 40,108,472 | 101,636 |
| SD | 99,918 | 99,918 | 25,045,210 | 25,043,287 | 61,977 |
| Median | 140,040 | 140,040 | 35,122,725 | 35,117,374 | 89,309 |
| Min | 46,981 | 46,981 | 11,776,954 | 11,775,592 | 27,243 |
| Max | 574,615 | 574,615 | 144,067,090 | 144,023,046 | 330,974 |

**Table S4:** Maximum values in the rarefaction curve

| **Sample ID** | **Maximum number of ASVs** | **Maximum sample size** | **Group** |
| --- | --- | --- | --- |
| S01 | 210 | 115099 | Control |
| S02 | 302 | 98568 | Control |
| S03 | 438 | 71437 | Control |
| S04 | 335 | 67007 | Control |
| S13 | 547 | 58814 | Control |
| S14 | 370 | 50066 | Control |
| S15 | 393 | 119165 | Control |
| S16 | 416 | 99055 | Control |
| S17 | 486 | 112930 | Control |
| S18 | 235 | 104074 | Control |
| S29 | 430 | 73016 | Control |
| S30 | 417 | 118992 | Control |
| S31 | 409 | 87275 | Control |
| S32 | 195 | 100545 | Control |
| S33 | 524 | 88608 | Control |
| S34 | 445 | 108837 | Control |
| S35 | 329 | 111094 | Control |
| S44 | 418 | 71296 | Control |
| S45 | 234 | 37442 | Control |
| S46 | 221 | 38681 | Control |
| S47 | 446 | 165402 | Control |
| S48 | 527 | 101119 | Control |
| S49 | 576 | 106640 | Control |
| S50 | 517 | 140731 | Control |
| S51 | 381 | 96650 | Control |
| S52 | 510 | 107359 | Control |
| S53 | 164 | 27243 | Control |
| S56 | 320 | 54061 | Control |
| S57 | 475 | 84021 | Control |
| S58 | 751 | 124256 | Control |
| S59 | 434 | 44492 | Control |
| S60 | 188 | 74329 | Control |
| S70 | 697 | 327916 | Control |
| S71 | 698 | 159999 | Control |
| S72 | 425 | 248161 | Control |
| S73 | 411 | 184686 | Control |
| S05 | 182 | 51047 | CLP |
| S06 | 296 | 47846 | CLP |
| S07 | 292 | 59532 | CLP |
| S08 | 219 | 80425 | CLP |
| S10 | 496 | 97139 | CLP |
| S11 | 334 | 86652 | CLP |
| S12 | 279 | 56090 | CLP |
| S19 | 276 | 45981 | CLP |
| S21 | 423 | 80941 | CLP |
| S22 | 681 | 131899 | CLP |
| S23 | 472 | 118480 | CLP |
| S24 | 342 | 90007 | CLP |
| S25 | 372 | 54733 | CLP |
| S26 | 199 | 37129 | CLP |
| S27 | 259 | 40657 | CLP |
| S36 | 429 | 160737 | CLP |
| S37 | 272 | 59547 | CLP |
| S38 | 334 | 64440 | CLP |
| S39 | 193 | 67649 | CLP |
| S40 | 476 | 85373 | CLP |
| S41 | 351 | 68491 | CLP |
| S42 | 272 | 90564 | CLP |
| S43 | 208 | 100372 | CLP |
| S54 | 518 | 143157 | CLP |
| S55 | 304 | 104727 | CLP |
| S61 | 296 | 72201 | CLP |
| S62 | 439 | 100002 | CLP |
| S63 | 386 | 52954 | CLP |
| S64 | 367 | 105379 | CLP |
| S65 | 229 | 82104 | CLP |
| S66 | 703 | 232868 | CLP |
| S67 | 331 | 330967 | CLP |
| S68 | 344 | 49886 | CLP |
| S69 | 298 | 285438 | CLP |

**Table S5:** Most abundant genera and their relative abundance regarding cleft lip and palate and control samples.

| **Genus** | **Mean in control (%)** | **SD in control (%)** | **Mean in CLP (%)** | **SD in CLP (%)** |
| --- | --- | --- | --- | --- |
| *Prevotella* | 15.56 | 6.29 | 20.03 | 6.77 |
| *Streptococcus* | 11.22 | 4.94 | 11.98 | 5.28 |
| *Haemophilus* | 2.84 | 3.78 | 4.02 | 5.59 |
| *Veillonella* | 8.62 | 3.10 | 8.43 | 3.73 |
| *Neisseria* | 9.67 | 6.58 | 7.23 | 5.51 |
| *Fusobacterium* | 5.12 | 3.12 | 5.41 | 2.98 |
| *Porphyromonas* | 5.08 | 5.06 | 4.42 | 4.04 |
| *Rothia* | 4.81 | 2.92 | 4.32 | 2.57 |
| *Gemella* | 3.59 | 3.35 | 2.75 | 2.11 |
| *Alloprevotella* | 2.87 | 1.86 | 2.88 | 2.60 |
| *Granulicatella* | 2.61 | 1.69 | 2.53 | 3.09 |
| *Nanosynbacter* | 2.36 | 2.40 | 2.64 | 2.17 |
| *Aggregatibacter* | 1.06 | 1.65 | 0.97 | 1.48 |
| *Lancefieldella* | 1.26 | 0.99 | 1.41 | 1.04 |
| *Oribacterium* | 1.07 | 0.68 | 0.95 | 0.78 |
| *Capnocytophaga* | 1.34 | 1.33 | 0.67 | 0.59 |
| *Pauljensenia* | 1.03 | 0.71 | 0.70 | 0.54 |

SD = Standard deviation. CLP = Cleft lip and palate

**Table S6:** Stratified comparison of alpha diversity between a group of mothers who had a child with cleft lip and palate (CLP) and a group of mothers who had a child with normal lip and palate (Control).

| **Only female** | Number of samples  Control = 14; CLP = 7 | | **Only male** | Number of samples  Control = 20; CLP = 23 | |
| --- | --- | --- | --- | --- | --- |
| Metric | H statistics | p-value | Metric | H statistics | p-value |
| Observed ASVs | 2.0093 | 0.1563 | Observed ASVs | 4.1840 | **0.0408*** |
| Evenness | 1.0909 | 0.2963 | Evenness | 1.8593 | 0.1727 |
| Simpson | 0.0501 | 0.8229 | Simpson | 2.5826 | 0.1080 |
| Shannon | 0.2004 | 0.6544 | Shannon | 3.7002 | 0.0544 |
| Chao1 | 2.4545 | 0.1172 | Chao1 | 4.2836 | **0.0385*** |
| Faith Phylogenetic Diversity | 1.6085 | 0.2047 | Faith Phylogenetic Diversity | 7.3049 | **0.0069*** |
| **Non-gingivitis** | Number of samples  Control = 21; CLP = 28 | | **Gingivitis** | Number of samples  Control = 13; CLP = 6 | |
| Metric | H statistics | p-value | Metric | H statistics | p-value |
| Observed ASVs | 3.0189 | 0.0823 | Observed ASVs | 1.3011 | 0.254 |
| Evenness | 0.5588 | 0.4548 | Evenness | 0.1923 | 0.661 |
| Simpson | 0.0690 | 0.7928 | Simpson | 0.0308 | 0.8608 |
| Shannon | 0.3200 | 0.5716 | Shannon | 1.3000 | 0.2542 |
| Chao1 | 2.6780 | 0.1017 | Chao1 | 1.1078 | 0.2926 |
| Faith Phylogenetic Diversity | 3.3061 | 0.06902 | Faith Phylogenetic Diversity | 2.2231 | 0.136 |
| **Only non-caries** | Control = 27; CLP = 22 | | **Only caries** | Control = 6; CLP = 12 | |
| Metric | H statistics | p-value | Metric | H statistics | p-value |
| Observed ASVs | 4.8006 | **0.0284*** | Observed ASVs | 1.0625 | 0.3026 |
| Evenness | 0.8549 | 0.3552 | Evenness | 1.4825 | 0.2234 |
| Simpson | 0.0582 | 0.8094 | Simpson | 0.7105 | 0.3993 |
| Shannon | 0.9309 | 0.3346 | Shannon | 1.7193 | 0.1898 |
| Chao1 | 4.9782 | **0.0257*** | Chao1 | 0.8772 | 0.3490 |
| Faith Phylogenetic Diversity | 3.8804 | **0.0488*** | Faith Phylogenetic Diversity | 3.8684 | **0.0492*** |
| **Non-hypertensive** | Control = 24; CLP = 29 | | **Hypertensive** | Control = 11; CLP = 5 | |
| Metric | H statistics | p-value | Metric | H statistics | p-value |
| Observed ASVs | 4.2229 | **0.0399*** | Observed ASVs | 1.1583 | 0.2818 |
| Evenness | 0.1408 | 0.7075 | Evenness | 0.0289 | 0.8651 |
| Simpson | 1.3078 | 0.2528 | Simpson | 0.0289 | 0.8651 |
| Shannon | 1.7484 | 0.1861 | Shannon | 0.7219 | 0.3955 |
| Chao1 | 4.3707 | **0.0366*** | Chao1 | 1.4150 | 0.2342 |
| Faith Phylogenetic Diversity | 4.9093 | **0.0267*** | Faith Phylogenetic Diversity | 2.6984 | 0.1004 |
| **No antibiotic usage** | Control = 24; CLP = 16 | | **Antibiotic usage** | Control = 11; CLP = 18 | |
| Metric | H statistics | p-value | Metric | H statistics | p-value |
| Observed ASVs | 3.7348 | 0.0533 | Observed ASVs | 1.6994 | 0.1924 |
| Evenness | 0.6410 | 0.4233 | Evenness | 1.0687 | 0.3012 |
| Simpson | 2.3056 | 0.1289 | Simpson | 0.5838 | 0.4448 |
| Shannon | 2.2226 | 0.1360 | Shannon | 0.1293 | 0.7192 |
| Chao1 | 3.5244 | 0.0605 | Chao1 | 1.8182 | 0.1775 |
| Faith Phylogenetic Diversity | 4.1738 | **0.0410*** | Faith Phylogenetic Diversity | 1.9414 | 0.1635 |
| **Without urinary infection** | Control = 17; CLP = 19 | | **Urinary infection** | Control = 17; CLP = 14 | |
| Metric | H statistics | p-value | Metric | H statistics | p-value |
| Observed ASVs | 5.7244 | **0.0167*** | Observed ASVs | 0.3546 | 0.5515 |
| Evenness | 2.7676 | 0.0962 | Evenness | 2.9133 | 0.0878 |
| Simpson | 5.8762 | **0.0153*** | Simpson | 2.2752 | 0.1315 |
| Shannon | 4.5749 | **0.0324*** | Shannon | 0.1576 | 0.6914 |
| Chao1 | 5.4244 | **0.0199*** | Chao1 | 0.3545 | 0.5516 |
| Faith Phylogenetic Diversity | 6.3462 | **0.0118*** | Faith Phylogenetic Diversity | 0.9848 | 0.3210 |

Significance was assessed by the Kruskal-Wallis rank sum test with p-value < 0.05. Only categorical variables with counts > 5 in all cells of the contingency table were considered for the alpha diversity comparison.

**Table S7:** PERMANOVA (adonis2 R package) of other beta diversities between a group of mothers who had a child with a cleft lip and palate and a group of mothers who had a child with a normal lip and palate. 999 permutations were used for the tests.

| **Stratified to only female children**  Formula used = (Urinary infection + Gingivitis + Caries + Gestational hypertension + Antibiotic usage + Child age) + Child lip and palate | | | | | |
| --- | --- | --- | --- | --- | --- |
| **Metric** | **SumOfSqs** | **R²** | **Residual R²** | **F** | **P-value** |
| Aitchison | 4981 | 0.06930 | 0.56311 | 1.2306 | 0.125 |
| Bray-Curtis | 0.2694 | 0.06629 | 0.54272 | 1.0994 | 0.310 |
| Jaccard | 0.3364 | 0.06831 | 0.56318 | 1.0916 | 0.186 |
| Unweighted Unifrac | 0.0755 | 0.08803 | 0.58705 | 1.3496 | 0.207 |
| Weighted Unifrac | 9023 | 0.08522 | 0.44942 | 1.7065 | 0.133 |
| **Stratified to only male children**  Formula used = (Urinary infection + Gingivitis + Caries + Gestational hypertension + Antibiotic usage + Child age) + Child lip and palate | | | | | |
| **Metric** | **SumOfSqs** | **R²** | **Residual R²** | **F** | **P-value** |
| Aitchison | 3721 | 0.02593 | 0.83131 | 1.0916 | 0.164 |
| Bray-Curtis | 0.2655 | 0.03082 | 0.81115 | 1.2540 | 0.081 |
| Jaccard | 0.3207 | 0.02650 | 0.81969 | 1.0669 | 0.174 |
| Unweighted Unifrac | 0.09221 | 0.04576 | 0.77338 | 1.9524 | **0.038*** |
| Weighted Unifrac | 9015 | 0.03526 | 0.81510 | 1.4274 | 0.207 |
| **Stratified into mothers with healthy gingiva**  Formula used = (Sex of the child + Urinary infection + Caries + Gestational hypertension + Antibiotic usage + Child age) + Child lip and palate | | | | | |
| **Metric** | **SumOfSqs** | **R²** | **Residual R²** | **F** | **P-value** |
| Aitchison | 3620 | 0.02433 | 0.82878 | 1.0275 | 0.351 |
| Bray-Curtis | 0.1763 | 0.02009 | 0.79984 | 0.8290 | 0.834 |
| Jaccard | 0.3162 | 0.02607 | 0.81730 | 1.0526 | 0.262 |
| Unweighted Unifrac | 0.06962 | 0.03466 | 0.78928 | 1.4493 | 0.167 |
| Weighted Unifrac | 6719 | 0.02942 | 0.72490 | 1.3394 | 0.241 |
| **Stratified into mothers with gingivitis**  Formula used = (Sex of the child + Urinary infection + Caries + Gestational hypertension + Antibiotic usage + Child age) + Child lip and palate | | | | | |
| **Metric** | **SumOfSqs** | **R²** | **Residual R²** | **F** | **P-value** |
| Aitchison | 5296 | 0.07847 | 0.54148 | 1.4492 | **0.004*** |
| Bray-Curtis | 0.4099 | 0.10667 | 0.47629 | 2.0157 | **0.001*** |
| Jaccard | 0.3507 | 0.07159 | 0.54499 | 1.1823 | **0.039*** |
| Unweighted Unifrac | 0.1067 | 0.12142 | 0.49730 | 2.1974 | **0.029*** |
| Weighted Unifrac | 16282 | 0.12260 | 0.39991 | 2.7592 | 0.074 |
| **Stratified into non-caries**  Formula used: (Sex of the child + Urinary infection + Gingivitis + Gestational hypertension + Antibiotic usage + Child age) + Child lip and palate | | | | | |
| **Metric** | **SumOfSqs** | **R²** | **Residual R²** | **F** | **P-value** |
| Aitchison | 4073 | 0.02634 | 0.82506 | 1.1495 | 0.102 |
| Bray-Curtis | 0.2289 | 0.02504 | 0.80739 | 1.0546 | 0.345 |
| Jaccard | 0.3304 | 0.02664 | 0.81999 | 1.1047 | 0.098 |
| Unweighted Unifrac | 0.0854 | 0.04272 | 0.78677 | 1.8460 | **0.045*** |
| Weighted Unifrac | 7396 | 0.03295 | 0.80746 | 1.3874 | 0.204 |
| **Stratified into caries**  Formula used: (Sex of the child + Urinary infection + Gingivitis + Gestational hypertension + Antibiotic usage + Child age) + Child lip and palate | | | | | |
| **Metric** | **SumOfSqs** | **R²** | **Residual R²** | **F** | **P-value** |
| Aitchison | 3348 | 0.05451 | 0.54021 | 0.9081 | 0.763 |
| Bray-Curtis | 0.2278 | 0.06499 | 0.50200 | 1.0356 | 0.401 |
| Jaccard | 0.3161 | 0.06796 | 0.53075 | 1.0243 | 0.356 |
| Unweighted Unifrac | 0.0694 | 0.07769 | 0.52754 | 1.1782 | 0.280 |
| Weighted Unifrac | 12535 | 0.09110 | 0.49872 | 1.4614 | 0.239 |
| **Stratified into non-hypertensive**  Formula used: (Sex of the child + Urinary infection + Gingivitis + Caries + Antibiotic usage + Child age) + Child lip and palate | | | | | |
| **Metric** | **SumOfSqs** | **R²** | **Residual R²** | **F** | **P-value** |
| Aitchison | 4104 | 0.02496 | 0.83614 | 1.1941 | 0.080 |
| Bray-Curtis | 0.2167 | 0.02326 | 0.80865 | 1.0642 | 0.318 |
| Jaccard | 0.3152 | 0.02407 | 0.82773 | 1.0760 | 0.168 |
| Unweighted Unifrac | 0.0802 | 0.03811 | 0.78922 | 1.7868 | 0.062 |
| Weighted Unifrac | 8732 | 0.03607 | 0.76468 | 1.7453 | 0.104 |
| **Stratified into hypertensive**  Formula used: (Sex of the child + Urinary infection + Gingivitis + Caries + Antibiotic usage + Child age) + Child lip and palate | | | | | |
| **Metric** | **SumOfSqs** | **R²** | **Residual R²** | **F** | **P-value** |
| Aitchison | 3803 | 0.07360 | 0.41750 | 0.8814 | 0.741 |
| Bray-Curtis | 0.2397 | 0.07382 | 0.40764 | 0.9054 | 0.609 |
| Jaccard | 0.3070 | 0.07806 | 0.42644 | 0.9153 | 0.761 |
| Unweighted Unifrac | 0.04636 | 0.05945 | 0.54488 | 0.7069 | 0.709 |
| Weighted Unifrac | 4971 | 0.04217 | 0.32308 | 0.6526 | 0.612 |
| **Stratified into patients without antibiotic usage**  Formula used: (Sex of the child + Urinary infection + Gingivitis + Caries + Gestational hypertension + Child age) + Child lip and palate | | | | | |
| **Metric** | **SumOfSqs** | **R²** | **Residual R²** | **F** | **P-value** |
| Aitchison | 4683 | 0.03641 | 0.77956 | 1.2611 | **0.038*** |
| Bray-Curtis | 0.2472 | 0.03419 | 0.76530 | 1.0722 | 0.285 |
| Jaccard | 0.3620 | 0.03805 | 0.76409 | 1.1952 | **0.033*** |
| Unweighted Unifrac | 0.1005 | 0.06366 | 0.74359 | 2.0548 | **0.023*** |
| Weighted Unifrac | 6475 | 0.03045 | 0.77599 | 0.9416 | 0.385 |
| **Stratified into patients with antibiotic usage**  Formula used: (Sex of the child + Urinary infection + Gingivitis + Caries + Gestational hypertension + Child age) + Child lip and palate | | | | | |
| **Metric** | **SumOfSqs** | **R²** | **Residual R²** | **F** | **P-value** |
| Aitchison | 3223 | 0.03707 | 0.70891 | 0.9413 | 0.703 |
| Bray-Curtis | 0.2723 | 0.05119 | 0.68276 | 1.3496 | 0.055 |
| Jaccard | 0.2819 | 0.03769 | 0.71240 | 0.9524 | 0.686 |
| Unweighted Unifrac | 0.0587 | 0.04643 | 0.70907 | 1.1787 | 0.258 |
| Weighted Unifrac | 12762 | 0.08674 | 0.62349 | 2.5042 | **0.022*** |
| **Stratified to only mothers without urinary infection**  Formula used = (Sex of the child + Gingivitis + Caries + Gestational hypertension + Antibiotic usage + Child age) + Child lip and palate | | | | | |
| **Metric** | **SumOfSqs** | **R²** | **Residual R²** | **F** | **P-value** |
| Aitchison | 4574 | 0.04133 | 0.74771 | 1.2713 | **0.046*** |
| Bray-Curtis | 0.2415 | 0.03812 | 0.72250 | 1.1079 | 0.250 |
| Jaccard | 0.3611 | 0.04227 | 0.72561 | 1.2233 | **0.030*** |
| Unweighted Unifrac | 0.1024 | 0.07424 | 0.65896 | 2.3659 | **0.030*** |
| Weighted Unifrac | 4754 | 0.02964 | 0.74018 | 0.8410 | 0.479 |
| **Stratified to only mothers with urinary infection**  Formula used = (Sex of the child + Gingivitis + Caries + Gestational hypertension + Antibiotic usage + Child age) + Child lip and palate | | | | | |
| **Metric** | **SumOfSqs** | **R²** | **Residual R²** | **F** | **P-value** |
| Aitchison | 3169 | 0.03002 | 0.74760 | 0.8834 | 0.888 |
| Bray-Curtis | 0.2445 | 0.03933 | 0.73811 | 1.1189 | 0.224 |
| Jaccard | 0.2702 | 0.03177 | 0.75220 | 0.8869 | 0.948 |
| Unweighted Unifrac | 0.0638 | 0.04181 | 0.72983 | 1.2030 | 0.252 |
| Weighted Unifrac | 10324 | 0.05556 | 0.74352 | 1.5694 | 0.156 |

**Table S8:** Number of differentially abundant taxonomies by stratified comparisons

| **All samples** | | |
| --- | --- | --- |
| **Taxonomic level** | **Number of taxa with adjusted p-value < 0.05** | **Differentially abundant taxa** |
| Phylum | 0 | - |
| Class | 1 | Alphaproteobacteria |
| Order | 1 | Sphingomonadales |
| Family | 0 | - |
| Genus | 1 | *Cutibacterium* |
| **Mothers of a male child** | | |
| **Taxonomic level** | **Number of taxa with adjusted p-value < 0.05** | **Differentially abundant taxa** |
| Phylum | 1 | Cyanobacteriota |
| Class | 1 | Cyanobacteriia |
| Order | 1 | Cyanobacteriales |
| Family | 2 | *Coleofasciculaceae* |
|  |  | *Lactobacillaceae* |
| Genus | 2 | *Caldora* |
|  |  | *Limosilactobacillus* |
| **Mothers without urinary infection** | | |
| **Taxonomic level** | **Number of taxa with adjusted p-value < 0.05** | **Differentially abundant taxa** |
| Phylum | 0 | - |
| Class | 0 | - |
| Order | 0 | - |
| Family | 0 | - |
| Genus | 0 | *-* |
| **Mothers with gingivitis** | | |
| **Taxonomic level** | **Number of taxa with adjusted p-value < 0.05** | **Differentially abundant taxa** |
| Phylum | 0 | - |
| Class | 0 | - |
| Order | 0 | - |
| Family | 0 | - |
| Genus | 0 | *-* |
| **Mothers without caries** | | |
| **Taxonomic level** | **Number of taxa with adjusted p-value < 0.05** | **Differentially abundant taxa** |
| Phylum | 0 | - |
| Class | 0 | - |
| Order | 1 | Sphingomonadales |
| Family | 0 | - |
| Genus | 1 | *Cutibacterium* |
| **Non-hypertensive mothers** | | |
| **Taxonomic level** | **Number of taxa with adjusted p-value < 0.05** | **Differentially abundant taxa** |
| Phylum | 0 | - |
| Class | 1 | Alphaproteobacteria |
| Order | 1 | Sphingomonadales |
| Family | 0 | - |
| Genus | 1 | *Cutibacterium* |
| **Mothers without antibiotic use** | | |
| **Taxonomic level** | **Number of taxa with adjusted p-value < 0.05** | **Found taxa** |
| Phylum | 0 | - |
| Class | 0 | - |
| Order | 0 | - |
| Family | 0 | - |
| Genus | 0 | *-* |

Stratified differential abundance analysis was performed only for variables that had at least three metrics of alpha or beta diversity with a statistically significant difference (p-value < 0.05).

**Table S9:** Number of categories of metabolic pathways (MetaCyc) with significant differences in enrichment between the CLP group and the control

| **Top-level description enriched in CLP** | **Second-level description enriched in CLP** | **Top-level description depleted in CLP** | **Second-level description depleted in CLP** |
| --- | --- | --- | --- |
| Biosynthesis (114) | -Cofactor, Prosthetic Group, Electron Carrier, and Vitamin Biosynthesis (33)  -Amino Acid Biosynthesis (23)  -Nucleoside and Nucleotide Biosynthesis (19)  -Cell Structure Biosynthesis (10)  -Carbohydrate Biosynthesis (10)  -Fatty Acid and Lipid Biosynthesis (9)  -Secondary Metabolite Biosynthesis (5)  -Aromatic Compound Biosynthesis (2)  -Amine and Polyamine Biosynthesis (1)  -Aminoacyl-tRNA Charging (1)  -Metabolic Regulator Biosynthesis (1) | No significant metabolic pathways | No significant metabolic pathways |
| Degradation / Utilization / Assimilation (20) | -Aromatic Compound Degradation (0)  -Amino Acid Degradation (2)  -Carboxylate Degradation (3)  -C1 Compound Utilization and Assimilation (4)  -Carbohydrate Degradation (4)  -Nucleoside and Nucleotide Degradation (3)  -Amine and Polyamine Degradation (1)  -Inorganic Nutrient Metabolism (1)  -Polymeric Compound Degradation (1)  -Secondary Metabolite Degradation (1) | Degradation / Utilization / Assimilation (7) | Aromatic Compound Degradation (5)  -Amino Acid Degradation (1)  -Carboxylate Degradation (1) |
| Generation of Precursor Metabolite and Energy (8) | -Glycolysis (4)  -Fermentation (3)  -Phytosynthesis (3)  -Pentose Phosphate Pathways (2) | No significant metabolic pathways | No significant metabolic pathways |
| Superpathways (2) | -Aspartate superpathway (1)  -Superpathway of S-adenosyl-L-methionine biosynthesis (1) | No significant metabolic pathways | No significant metabolic pathways |
| Macromolecule Modification (2) | Nucleic Acid Processing (2) | No significant metabolic pathways | No significant metabolic pathways |
| Glycan Pathways (1) | Glycan Biosynthesis (1) | No significant metabolic pathways | No significant metabolic pathways |

**Table S11:** Number of categories of metabolic pathways (MetaCyc) of mothers of only male children upregulated in the CLP group compared to the control group (p-value < 0.05).

| **Top-level description enriched in CLP** | **Second-level description enriched in CLP** | **Top-level description depleted in CLP** | **Second-level description depleted in CLP** |
| --- | --- | --- | --- |
| Biosynthesis (125) | -Cofactor, Prosthetic Group, Electron Carrier, and Vitamin Biosynthesis (39)  -Amino Acid Biosynthesis (27)  -Nucleoside and Nucleotide Biosynthesis (19)  -Cell Structure Biosynthesis (10)  -Carbohydrate Biosynthesis (10)  -Fatty Acid and Lipid Biosynthesis (9)  -Secondary Metabolite Biosynthesis (6)  -Aromatic Compound Biosynthesis (2)  -Amine and Polyamine Biosynthesis (1)  -Aminoacyl-tRNA Charging (1)  -Metabolic Regulator Biosynthesis (1) | No significant metabolic pathways | No significant metabolic pathways |
| Degradation/Utilization/Assimilation (32) | -Aromatic Compound Degradation (0)  -Amino Acid Degradation (2)  -Carboxylate Degradation (3)  -Carbohydrate Degradation (7)  -C1 Compound Utilization and Assimilation (4)  -Nucleoside and Nucleotide Degradation (3)  -Inorganic Nutrient Metabolism (2)  -Secondary Metabolite Degradation (2)  -Amine and Polyamine Degradation (1)  -Polymeric Compound Degradation (1) | Degradation/Utilization/Assimilation (7) | -Aromatic Compound Degradation (5)  -Amino Acid Degradation (1)  -Carboxylate Degradation (1) |
| Generation of Precursor Metabolite and Energy (14) | -Glycolysis (4)  -Fermentation (3)  -Phytosynthesis (3)  -Pentose Phosphate Pathways (2)  -TCA cycle (1)  -Others(1) | No significant metabolic pathways | No significant metabolic pathways |
| Superpathways (2) | -Aspartate superpathway (1)  -Superpathway of S-adenosyl-L-methionine biosynthesis (1) | No significant metabolic pathways | No significant metabolic pathways |
| Macromolecule Modification (2) | Nucleic Acid Processing (2) | No significant metabolic pathways | No significant metabolic pathways |
| Glycan Pathways (1) | Glycan Biosynthesis (1) | No significant metabolic pathways | No significant metabolic pathways |
